# The *WWOX* Gene Influences Cellular Pathways in the Neuronal Differentiation of Human Neural Progenitor Cells

**DOI:** 10.3389/fncel.2019.00391

**Published:** 2019-08-30

**Authors:** Katarzyna Kośla, Elżbieta Płuciennik, Ewa Styczeń-Binkowska, Magdalena Nowakowska, Magdalena Orzechowska, Andrzej K. Bednarek

**Affiliations:** Department of Molecular Carcinogenesis, Medical University of Łódź, Łódź, Poland

**Keywords:** *WWOX*, neural progenitor cells, neuronal differentiation, neurodegeneration, WOREE, SCAR, CAGE

## Abstract

The brain is the most functionally organized structure of all organs. It manages behavior, perception and higher cognitive functions. The *WWOX* gene is non-classical tumor suppressor gene, which has been shown to have an impact on proliferation, apoptosis and migration processes. Moreover, genetic aberrations in *WWOX* induce severe neuropathological phenotypes in humans and rodents. The aim of the present study was to investigate in detail the impact of *WWOX* on human neural progenitor cell (hNPC) maintenance and how depletion of *WWOX* disturbs signaling pathways playing a pivotal role in neuronal differentiation and central nervous system (CNS) organogenesis. hNPC with a silenced *WWOX* gene exhibited lowered mitochondrial redox potential, enhanced adhesion to fibronectin and extracellular matrix protein mixture, downregulation of MMP2/9 expression and impaired 3D growth. Global transcriptome analysis using cap analysis of gene expression (CAGE) found that *WWOX* downregulation significantly changes the expression of multiple genes engaged in cytoskeleton organization, adhesion, cell signaling and chromatin remodeling. The massive changes in gene expression caused by *WWOX* silencing may strongly affect the differentiation and migration of neurons in organogenesis, brain injury, cancerogenesis or neurodifferentiation. *WWOX* gene appears to be an important regulator of neural tissue architecture and function.

## Introduction

The *WWOX* gene is an important transcription regulator. It is known to regulate the activity of a number of transcription factors, including Jun, AP2gamma, NFkappaB, ErbB4 (Aqeilan et al., 2004a, 2005; Gaudio et al., 2006; Chen et al., 2013). It has been found to have tumor suppressor activity, and recent studies have investigated its potential new physiological roles in DNA repair and glucose metabolism (Aqeilan et al., 2005; Abu-Remaileh and Aqeilan, 2014; Iatan et al., 2014). In addition to steroid-hormone regulated tissues like those in the testis, ovary and breast, the *WWOX* gene also shows high expression in the brain and cerebellum (Nunez et al., 2006). Loss of correct *WWOX* expression is a common event associated with cancer promotion, progression and resistance to treatment (Płuciennik et al., 2006, 2014; Dias et al., 2007; Donati et al., 2007; Wang et al., 2011; Lan et al., 2012; Sun et al., 2017; Seabra et al., 2018). Unlike classic suppressor genes, the loss of functionality of only one *WWOX* allele is sufficient to increase the chance of cancerogenesis (haploinsufficiency).

WWOX has also been found to play a significant role in glioblastoma (GBM), the most malignant brain tumor. It has been shown that loss of heterozygosity, a relatively frequent occurrence in GBM, along with promoter methylation may decrease the expression of the *WWOX* gene. Our experiment also revealed a correlation between the expression of *WWOX* and that of *Bcl2*, *Ki67*, and *ErbB4* in this type of cancer (Kosla et al., 2011). Further investigations have found *WWOX* expression to modulate the global profile of gene expression in the GBM T98G cell line, and increased *WWOX* expression makes its phenotype less malignant. Cells with ectopically expressed *WWOX* demonstrate significantly different transcription profiles among almost 3000 genes, with WNT, TGFβ, Notch and Hedgehog being the main cellular pathways affected; all of these are involved both in GBM carcinogenesis and neural differentiation. Moreover, the *WWOX*-transfected GBM cells presented greatly altered biological characteristics, lowered proliferation, adhesion and impaired 3D growth (Kośla et al., 2014).

Recent evidence suggests that *WWOX* may play a regulatory role in central nervous system (CNS) development and functioning (Chen et al., 2004; Lo et al., 2008; Suzuki et al., 2009; Tabarki et al., 2015a; Liu et al., 2018; Shaukat et al., 2018; Hussain et al., 2019). Complete loss of WWOX protein function in knock-out mice results in epilepsy and balance disturbances. The animals show progressive susceptibility to spontaneous and audiogenic tonic-clonic epilepsy, which is indicative of a neurodegenerative process (Mallaret et al., 2014). Suzuki et al. (2009) report the presence of a spontaneous homozygous 13-bp deletion in exon 9 of the *WWOX* gene that has also been found in *Ide/Ide* rats. This deletion results in a frameshift in the C-terminus of the WWOX protein and may influence protein stability, since WWOX protein was undetectable in the hippocampus of the *Ide/Ide* rats. In addition to various abnormalities, such as dwarfism, postnatal lethality and male hypogonadism, the animals also display a high incidence of epilepsy, ataxic gait, audiogenic seizures and many vacuoles in the hippocampus and amygdala, indicating the progression of neurodegeneration (Suzuki et al., 2009). There is a strong overlap between the symptoms present in KO *WWOX* rodent models and those observed in humans. The genetic aberrations in the *WWOX* locus has been linked with two human neuropathological phenotypes: Spinocerebellar ataxia, autosomal recessive 12 (SCAR12, MIM 614322) and WWOX-Related Epileptic Encephalopathy/Early Infantile Epileptic Encephalopathy 28 (WOREE/EIEE28, MIM 616211). All known patients suffering from SCAR12 carry a homozygous missense mutation in *WWOX* coding region (139C > A or 1114G > C) that causes a partial loss of gene function, with the resulting substitutions placed in the WW1 protein–protein interaction domain and in the SDR (short-chain dehydrogenase/reductase) domain, respectively. These mutations affect the ability of the protein to bind to its cellular partners and may reduce its catalytic activity. The WOREE patients exhibiting more severe neurological disorders harbor nonsense/frameshift mutations and/or robust deletions of whole exons of *WWOX* gene (Mignot et al., 2015; Tabarki et al., 2015b; Elsaadany et al., 2016; Piard et al., 2018).

Overall, reports describing neural aberrations connected with disruption of *WWOX locus* in humans and animals indicate that germline loss-of-function of *WWOX* leads to serious developmental deficiency in the neural system.

The *WWOX* gene was found to be also involved in neurodegenerative Alzheimer’s disease. The level of WWOX protein is much lower in the hippocampal neurons of those suffering from Alzheimer’s disease in comparison to healthy individuals (Sze et al., 2004; Teng et al., 2012). Moreover, *in vitro* studies of *WWOX* silencing in neuronal cells have resulted in spontaneous Tau phosphorylation and accumulation of neurofibrillary tangles (NFTs). It has been confirmed that WWOX interacts with GSK3β, ERK and other kinases inhibiting the phosphorylation of Tau and preventing NFTs formation and cell death. The role of WWOX as a Tau phosphorylation regulator is important not only in the context of neurodegeneration, but also neuronal differentiation, where the Tau protein takes part in microtubule assembly and neurite outgrowth. The level of *WWOX* expression was shown to decrease with age and is significantly lower in middle-aged individuals (Sze et al., 2004; Wang et al., 2012).

To date, WWOX has been experimentally shown to act as a regulator of several transcription factors, including p73 (considered as one of the main regulators of CNS organogenesis), ErbB4, Met, c-Jun, Gli1, and AP-2 (Aqeilan et al., 2004a, b, 2005; Gaudio et al., 2006; Matteucci et al., 2009; Xiong et al., 2014). The shared mechanism of regulation is based on partner protein sequestration in the cytoplasm. Upon binding to WWOX, the locked transcription factors are unable to translocate to the nucleus and activate their target genes. A set of 240 proteins thought to be able to bind with WWOX has been given by Abu-Odeh et al. (2014). An analysis of potential WWOX partners shows that the protein can affect pathways critical for neuronal differentiation, such as Notch, Hippo, Wnt, and TGFβ, which are also disturbed in the course of brain cancer.

So far, the molecular mechanism by which WWOX regulates nerve cell functioning is largely unknown. It has been suggested that *WWOX* may influence the CNS by regulating the proteins taking part in neuronal differentiation, through negative regulation of GSK3β, and apoptosis, probably via the mitochondrial pathway by downregulating apoptotic inhibitors Bcl2 and Bcl-xL. WWOX has also been shown to influence the action of such neural pathways as Wnt (Dvl-2), TGFβ (TIAF1, SMAD3, and SMAD7), Hedgehog (Gli1) by interacting with their constituent proteins (Bouteille et al., 2009; Ferguson et al., 2013; Xiong et al., 2014; Chiang et al., 2015; Hsu et al., 2016). A recent study on WWOX KO mice by Hussain et al. (2019) found that lack of the protein leads to a reduction of GABA-ergic hippocampal neurons and a lowered level of GABA synthesis.

The described neuropathological phenotypes arising from genetic aberrations of the *WWOX* locus demonstrates the importance of the gene in CNS development and functioning. Although *WWOX* mutations found in SCAR12 or WOREE are very rare, possibly because of intrauteral lethality, changes in its expression are very common in pathological states like Alzheimer’s disease or GBM.

The development of the CNS is a highly organized process consisting of neurogenesis, neuron migration, synaptogenesis and gliogenesis, which is controlled at multiple levels. It is likely that the WWOX protein is involved in at least some of these steps. Briefly, the process of cytoskeleton reorganization requires a complex configuration of intrinsic and extrinsic cues involving the action of membrane and ECM proteins, which triggers specific signaling pathways. Its details are still poorly understood and await further investigation. Our findings indicate that the silencing of WWOX in human neural progenitors results in significant changes in the regulation of genes encoding membrane/ECM, cell signaling, chromatin condensation and cytoskeleton proteins.

The study examines which specific genes and cellular pathways are influenced by WWOX in human neural progenitor cells (hNPC) and how *WWOX* affects their biological properties and differentiation. *WWOX* expression was silenced in the hNPC line, and the cells were tested for their ECM adhesion ability, mitochondrial potential and 3D growth; in addition, a global gene expression analysis was conducted with next generation sequencing (NGS).

## Materials and Methods

### Cell Line and Culture Conditions

The human neural stem cells (hNSC, Thermo Fisher Scientific) are derived from H9 embryonic stem cells (hESC). According to manufacturer description they have the potential to differentiate into neurons, astrocytes and oligodendrocytes. The cells were cultured as an adherent culture in vessels coated with ECM protein mixture (Geltrex, Thermo Fisher Scientific). The cells were maintained in KnockOut^TM^ DMEM/F-12 Medium supplemented with 20 ng/ml EGF, FGFb, StemPro Neural Supplement (Thermo Fisher Scientific) and Antibiotic-Antimycotic (Gibco) and handled according to the supplier recommendations.

Spontaneous differentiation of the cells was triggered by culturing in medium without the addition of EGF and FGFb growth factors for 14 days. Although the supplier claims that the cells are able to differentiate into neurons, astrocytes and oligodendrocytes upon growth factor removal, in our conditions all the cells were found to undergo differentiation into neurons, which was confirmed by immunocytochemistry. All differentiated cells were found to be positive for neuronal markers MAP2 and TUJ1 and negative for astrocytes – GFAP or oligodendrocytes – GalC markers (Supplementary Figure S2). This suggests that the cells lose their multipotence and instead of being neural stem cells are rather a neural progenitors (hNPC) and were regarded in this study as such.

The 2D differentiation assays were conducted on Geltrex coated vessels, and neuron maturity was assessed by immunocytochemistry with antibodies against neuronal marker MAP2. For a 3D culture assay, 16,000, 25,000, and 35,000 cells were seeded on a solidified 2 mm layer of growth factor-reduced Geltrex basement membrane matrix (Thermo Fisher Scientific).

In every conducted experiment the cells from both experimental variants were in the same, low passage number.

### *WWOX* Gene Silencing

The *WWOX* gene was silenced by a shRNA lentiviral delivery system (Santa Cruz Biotechnology). The control cells were transduced with the same type of vector harboring scrambled shRNA sequence instead of targeting investigated gene. Target cells were seeded at a density of 5 × 10^4^ cells/cm^2^, and the next day, were infected with the virus suspension in culturing medium supplemented with polybrene 5 μg/ml (Sigma-Aldrich). After 24 h, the transduction mixture was removed and replaced with regular medium. The stable transductants were selected with 0.4 μg/ml puromycin. The gene silencing efficiency was assessed with Western blot.

### Western Blot

The cellular proteins were extracted with RIPA buffer supplemented with protease inhibitor cocktail, PMSF and Na-orthovanadate (Santa Cruz Biotechnology). Briefly, 60 μg of protein was resolved on SDS-PAGE and transferred to PVDF membrane. After blocking in 5% non-fat milk, membranes were incubated overnight in 4°C with a primary antibody, anti-WWOX (Thermo Fisher Scientific). After 1-h incubation with a suitable secondary antibody conjugated with alkaline phosphatase (Sigma-Aldrich), the membranes were developed with Novex AP Chromogenic Substrate (Thermo Fisher Scientific). The ImageJ software was used for densitometric analysis of protein amount, adjusted to GAPDH as a reference protein.

### Immunocytochemistry

The cells were fixed with ice cold ethanol:acetic acid (95:5) solution for 10 min. After a double wash with DPBS, unspecific antibody binding was blocked with blocking buffer (2% BSA, 1% donkey serum, and 0.1% Triton-X100). Next step was incubation with the primary antibodies (Millipore, Thermo Fisher Scientific) 1:200 solution in 5% donkey serum, 4°C, overnight. After double washing with DPBS, the cells were incubated with secondary antibodies (donkey, Alexa Fluor 488/594 conjugated), diluted 1:1000. The nuclei were counterstained with DAPI and the cells were imagined with FLoid Cell Imaging System (Thermo Fisher Scientific).

### Adhesion Assays

The cells were seeded on 96-well plate coated with Geltrex (Gibco) at a density of 17,500 cells/well. The adhesion to fibronectin was assessed on 48-well fibronectin-coated plate (BD). The cells were seeded at a density of 100,000 cells/well. The cells were incubated for 90 min at 37°C. Next the cells were washed with PBS and adherent cells were stained with Cell Stain Solution (Cell Biolabs, Inc.). The stain was dissolved in Extraction Solution (Cell Biolabs, Inc.) and the absorbance was read with a plate reader (BioTek), λ = 570 nm.

### Zymography

The level of metalloproteinase 2 and 9 was examined by gelatin zymography. The cells were cultured on a six-well plate for 48 h and the culture medium was collected for protein analysis. Protein concentration was measured with a Qubit Protein Assay with Qubit 2.0 fluorometer (Thermo Fisher Scientific). A total of 2 μg protein extracts was loaded and separated on 10% SDS-PAGE gel prepared with addition of 2 mg/ml gelatin. The gel was washed with Triton X-100 and incubated in a developing buffer (0.5 M Tris–HCl, 2 M NaCl, 50 mM CaCl 2, pH 7.5) at 37°C for 18 h. Next, it was stained with Coomassie Brilliant Blue R-250 and incubated in destaining buffer (methanol: acetic acid: water, 3:1:6) to visualize white bands on a blue background where gelatinolytic metalloproteinases were active. The active or inactive form of MMP2 and 9 was identified according to protein marker separated on the same gel. The amount of the proteins was assessed with ImageJ software, based on the area of the clear band.

### Redox Assay

The mitochondrial metabolic activity was measured using PrestoBlue reagent (resazurin based, Thermo Fisher Scientific). Cells were seeded on 96-well white, clear-bottom 96-well plate at a density of 20,000 cells/well. PrestoBlue was added to fresh medium 18 h post seeding and the cells were incubated in 37°C. The fluorescence of resorufin obtained by reduction of resazurin in mitochondria was measured in intervals for 5 h with Victor X4 plate reader (PerkinElmer), excitation λ = 550 nm.

### Statistical Analysis of Biological Assays

The results of the adhesion, mitochondrial redox potential and zymography procedures are presented as means. The normality of data distribution was verified with Shapiro–Wilk Normality Test and the statistical relevance was evaluated with unpaired *t*-test. The results are described as significant when *p* < 0.05.

### CAGE Gene Expression Analysis

The gene expression profile of 2D cultured undifferentiated and differentiated cells were analyzed with cap analysis of gene expression (CAGE) technique.

The libraries for sequencing were prepared according to Carnici, described in detail elsewhere (Takahashi et al., 2012). Briefly, polyadenylated and non-polyadenylated RNA was reverse transcribed into cDNA with the use of a random prime-containing *Eco*P15I sequence. The next step was biotinylation of the cap site and the 3′ ends of the hybridized RNAs. Non-hybridized ssRNAs were digested and the 5′ complete cDNAs hybridized to biotin-tagged, cap-containing RNAs were separated on streptavidin-coated magnetic beads. cDNA was released from RNA and a double-stranded 5′ linker with a barcode and *Eco*P15I sequences was ligated. Subsequently, using the biotinylated 2nd SOL primer, a second strand cDNA was synthetized. The digestion with *Eco*P15I allowed 27 bp fragments to be cleaved inside the 5′ end of the cDNA. At the 3′ end of the cDNA, a 3′ linker with 3′ Illumina primer sequence was ligated. Following this, the constructed CAGE tags were amplified and purified. The prepared libraries were sequenced on an Illumina HiSeq System. The data was then analyzed in-house. The raw data from CAGE experiment are deposited in NCBI Gene Expression Omnibus (GEO) Database with accession number GSE126075.

### Data Processing

The obtained CAGE data was processed by the MOIRAI system (Hasegawa et al., 2014), a GUI-based workflow system allowing quality control and further data transformation into multiple file formats for subsequent expression analyses. Briefly, raw input was split by barcode, following which, sequences of low quality (with base N) were removed and processed via BWA to output BAM files. Primarily, the GRCh37/hg19 assembly was used as a reference human genome; however, the data was reprocessed through UCSC liftOver tool (Kuhn et al., 2013) to convert the genomic coordinates of CAGE peaks into current GRCh38/hg38 assembly and make the data meaningful for the recent genome version. Subsequently, CAGE output was analyzed with CAGEr (Haberle et al., 2015), a Bioconductor package enabling preprocessing, identification and normalization of transcription start sites (TSS) and downstream analysis of the promoterome based upon CAGE sequencing with the BSgenome.Hsapiens.UCSC.hg38 package as a referent genome (Pagés, 2018). Analysis was performed according to Haberle and Plessy^1^. In brief, raw CAGE tag counts were normalized with simple TPM method, TSS were clustered into tag clusters corresponding to individual promoters using paraclu clustering algorithm and mapped to known promoters using CAGE peaks identified as true TSS by TSS classifier available at FANTOM5 repository (Lizio et al., 2015) (GRCh38/hg38 assembly).

### Global Biological Differentiation – Gene Set Enrichment Analysis

Global differences between hNPC with silenced *WWOX* expression and controls before and after differentiation were determined in terms of gene ontology (GO), such as biological processes (BP), by applying the gene set enrichment analysis (GSEA) algorithm. Enrichment analysis was performed for 9427 genes between shWWOX and shScrambled, classified as phenotype labels separately for hNPC and neurons through *t*-test with a weighted scoring scheme and a permutation type regarding phenotype. Significance threshold was set as FDR < 0.25.

### Protein–Protein Interaction Analysis

The interaction relationships of the proteins encoded by the differentially expressed genes were searched and visualized with the Search Tool for the Retrieval of Interacting Genes (STRING) Plug-in (version 1.4.0)^2^ in Cytoscape software (version 3.6.1)^3^. All the parameters were set as default values, and the confidence (combined score) ≥ 0.4 was used as the cut off criterion.

### Principle Component Analysis

Dimensional grouping investigating hNPC phenotypes according to a set of selected genes was performed using principle component analysis (PCA). The analysis involved 362 genes of several oxidative stress response/antioxidant defense/oxygen species activity connected groups form The Molecular Signatures Database (MSigDB)^4^, a collection of annotated gene sets. The PCA was conducted to determine the contribution of particular genes into partitioning of cell genotype across first and second dimensions (dim1 and dim2) with the oxidative gene set as an active variable. All analyses were performed using FactoMineR and Factoextra R packages.

## Results

**FIGURE 1:**
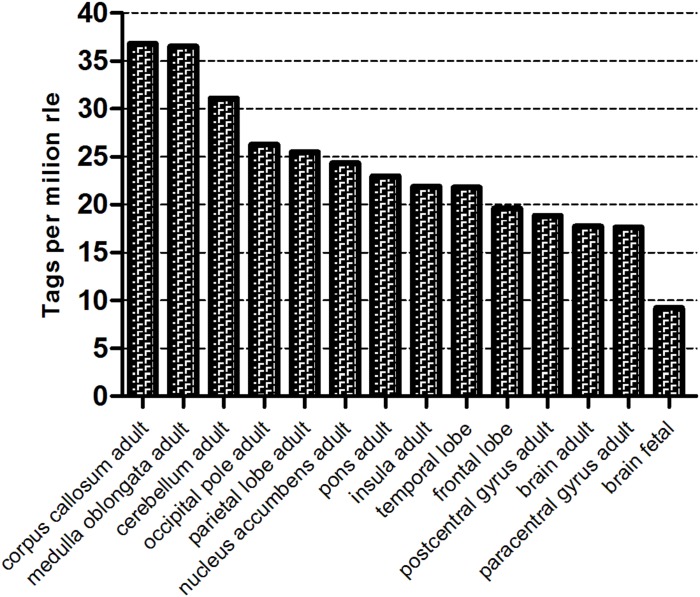
The *WWOX* gene expression level in different human brain compartment (Fantom 5, Zenbu, Riken). The counts were normalized as TPM (tags per million) after scaling by normalization factors calculated by RLE (relative log expression) method.

An analysis of NGS data deposited in the RIKEN Fantom5 Project data repository^5^ indicates that the level of *WWOX* expression in the human brain varies according to location (Figure 1). The highest levels are observed in the corpus callosum and medulla oblongata, and the lowest in the postcentral and paracentral gyrus. Moreover, adults display greater *WWOX* expression in the brain as a whole than 20–33 weeks fetuses, 17.7 TPM RLE (tags per million, relative log expression) vs. 9.2 TPM RLE.

In the present study, the hNPC cells were transduced with lentiviral shRNA vector. After confirmation of *WWOX* gene silencing by Western blot (Supplementary Figure S1) the hNPC/shWWOX cells and control hNPC/shScrambled were cultured either as a 2D monolayer culture using plates coated with a thin layer of diluted Geltrex or as a 3D culture in a thick (2–3 mm) Geltrex scaffold. Next, the cells were used in a number of biological experiments, and global analysis of transcriptome was performed using CAGE.

### *WWOX* Depletion Alters the Main Biological Functions of hNPC

A number of biological differences were observed between the hNPC cells with silenced *WWOX* and controls.

**FIGURE 2:**
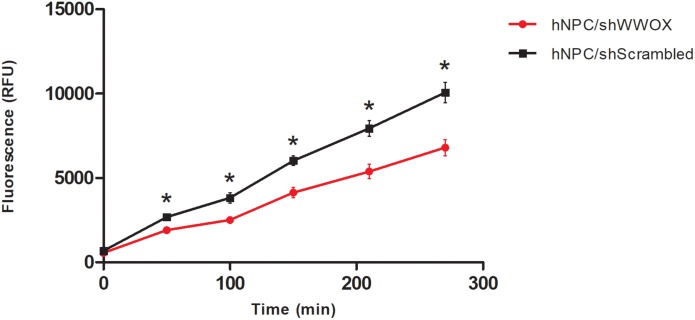
Mitochondrial redox activity tested by PrestoBlue^®^ assay based on colorimetric measurement of resofurin, ^∗^*p* < 0.05.

**FIGURE 3:**
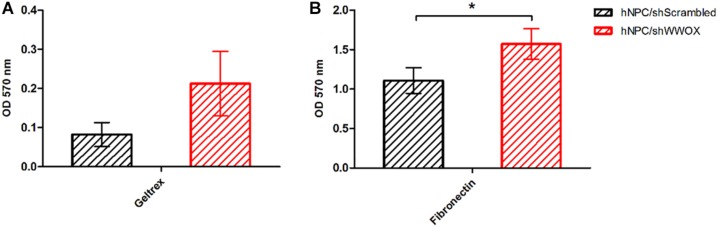
Adhesion of *WWOX* silenced hNPC cells to ECM protein mixture (Geltrex, **A**), the tendency not reaching the statistical significance (0.0626) and to fibronectin **(B)**, ^∗^*p* < 0.05.

*WWOX* silencing reduced the mitochondrial redox activity of the cells (Figure 2) and strongly affected their adhesion to ECM proteins. The hNPC cells with silenced *WWOX* demonstrated considerably stronger adhesion to the ECM protein mixture (*p* = 0.0626) and to fibronectin alone (*p* < 0.05) (Figure 3).

**FIGURE 4:**
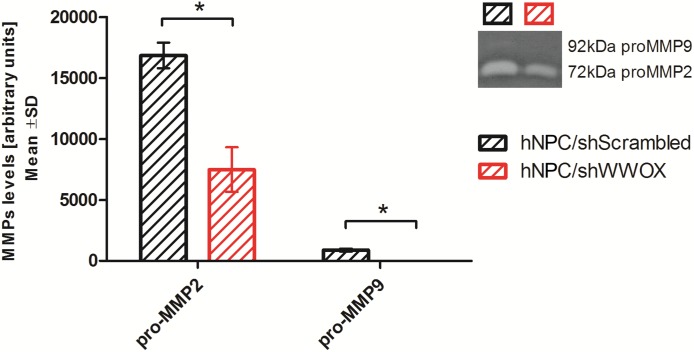
Gelatin zymography analysis showing the level of pro-MMP2 (72 kDa) and pro-MMP9 (92 kDa) secreted by control and WWOX-silenced hNPC, *^∗^p* < 0.05.

It was also found that downregulation of *WWOX* lowered pro-MMP2 and pro-MMP9 metalloproteinase secretion (Figure 4).

**FIGURE 5:**
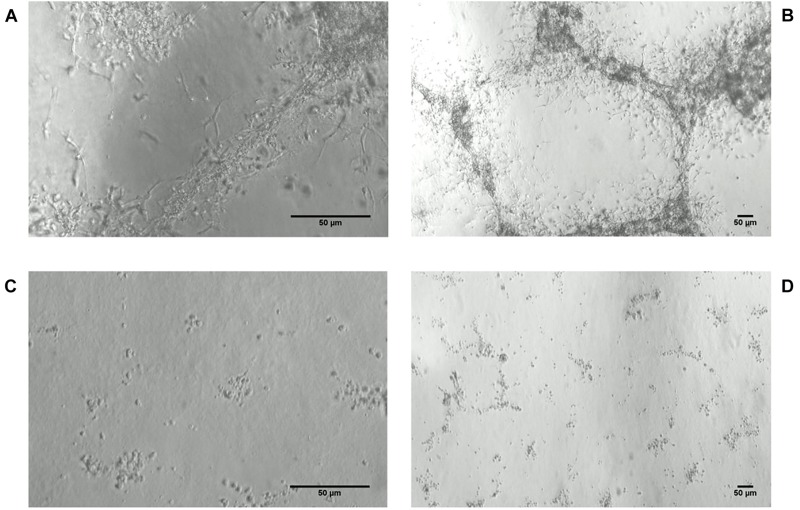
hNPC cells cultured for 8 days in thick 3D Geltrex (ECM protein mixture) layer. **(A,B)** hNPC/shScrambled, 200× and 40×, respectively. **(C,D)** hNPC/shWWOX, 200× and 40×, respectively.

**FIGURE 6:**
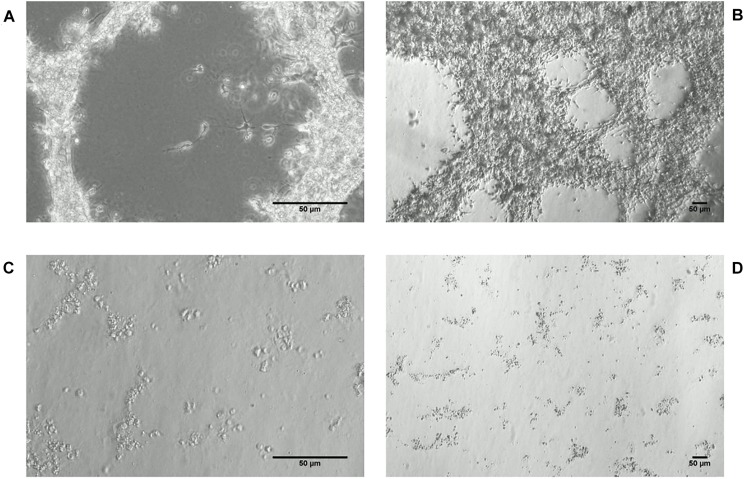
hNPC cells cultured for 19 days in thick 3D Geltrex (ECM protein mixture) layer. **(A,B)** hNPC/shScrambled, 200× and 40×, respectively. **(C,D)** hNPC/shWWOX, 200× and 40×, respectively.

**FIGURE 7:**
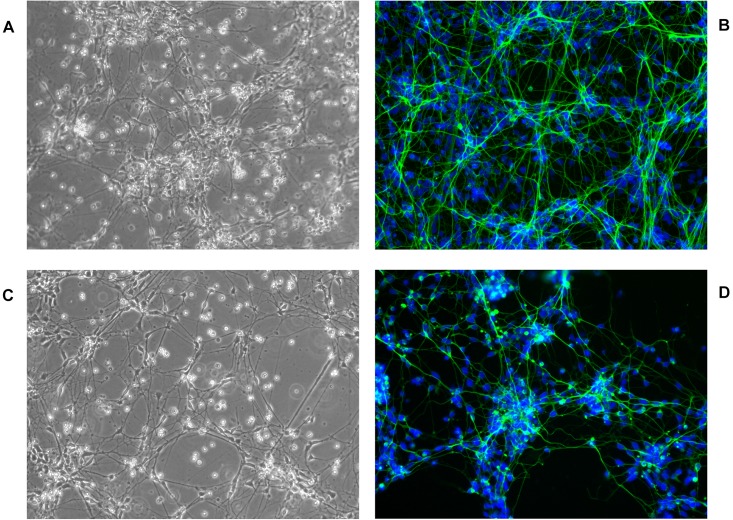
hNPC cells cultured for 18 days in 2D ECM protein mixture coated vessels, 200×. **(A,B)** hNPC/shScrambled, **(C,D)** hNPC/shWWOX, green MAP2 neuronal marker, blue DAPI nucleus counterstain.

Most striking was that in 3D culture, the hNPC with silenced *WWOX* remained as isolated, single cells that did not proliferate nor differentiate, while cells with unaltered *WWOX* expression differentiated and exhibited extensive network formation (Figures 5, 6). This observation was confirmed in three separate experiments and was not influenced by the seeding density (data not shown). No such phenomenon was observed when the cells were cultured in 2D as a monolayer (Figure 7).

### Functional Enrichment Analysis

**FIGURE 8:**
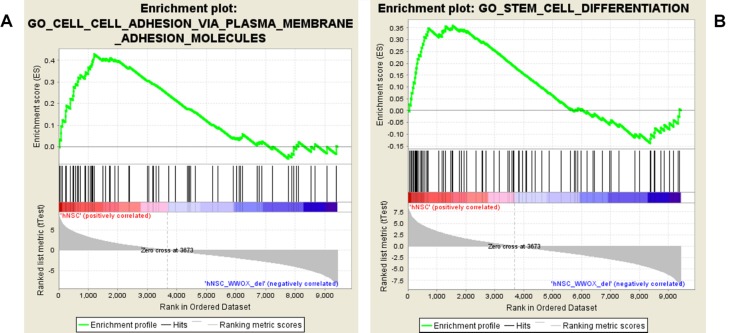
GSEA enrichment plots presenting adhesion **(A)** and differentiation **(B)** gene sets enriched in hNPC cells.

The global gene expression was assessed in the hNPC with the silenced *WWOX* expression and the control group before and after differentiation by CAGE. Whole transcriptome sequencing revealed global changes in the regulation of a number of pivotal cellular pathways and functions. It was found that 2282 genes were differentially expressed between hNPC/shScrambled and hNPC/sh*WWOX* (log2 FC > ±1, *p* < 0.05) and 7392 differed between differentiated neurons/shScrambled and neurons/sh*WWOX*. To identify global biological changes between cells with different *WWOX* status, enrichment analysis was performed within GO BP using a GSEA algorithm. The top five most altered BP classified according to GO terms are presented in Table 1 and includes adhesion, neural migration and differentiation. Significantly greater numbers of enriched gene sets was found for undifferentiated cells (44 for hNPC/shScrambled and 109 for hNPC/sh*WWOX*, FDR < 0.25) than for differentiated neurons (no gene sets for neurons/shScrambled and four sets in neurons/sh*WWOX*). The detailed results are available as Supplementary Tables S2, S3. Enrichment plots for selected gene sets are given in Figure 8.

**TABLE 1:** Top gene sets enriched in hNPC with native *WWOX* expression level.

**Gene set name**	**Gene number**	**Enrichment score**
GO_neural_crest_cell_differentiation	33	0.622
GO_neural_crest_cell_migration	21	0.677
GO_homophilic_cell_adhesion_via_ plasma_membrane_adhesion_molecules	53	0.511
GO_cell_cell_adhesion_via_plasma _membrane_adhesion_molecules	69	0.428
GO_negative_regulation_of_ axon_extension	21	0.578

*FDR *q*-value < 0.25.*

The GSEA showed that *WWOX* silencing causes substantial gene expression reorganization. We found dualistic enrichment of GO_Stem_Cell_Differentiation gene set in both phenotypes (Figure 8B). In the hNPC/shScrambled the leading edge subset (considered as the most upregulated and promising genes) included LOXL3, JAG1, SOX21, ERBB4, KITLG, GPM6A, EFNB1, FGF10, NRG1, WWTR1, RUNX2, LAMA5, MSI2, SEMA4D, BCHE, FOXC1, SMAD4, SEMA5A, SEMA5B, SEMA6C, SEMA3A, SEMA6A, SEMA3C, GSK3B, BMP7, SFRP1, SEMA4F, and SEMA4B, whereas the hNPC/sh*WWOX* leading edge subset included HHEX, FGFR2, GBX2, SNAI1, GREM1, MSX2, and MSX1.

### Protein–Protein Interaction Network Analysis

**FIGURE 9:**
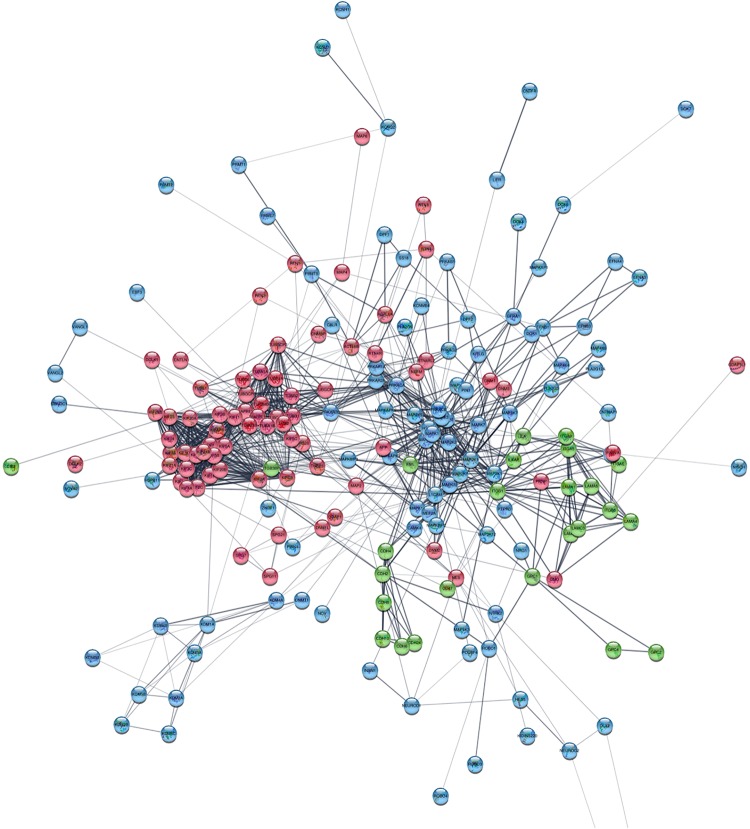
Protein–protein interaction network of differentially expressed genes involved in neuron migration. Cytoskeleton and ER are red; membrane and ECM proteins are green; cell signaling and chromatin remodeling are blue. High resolution figure with legible gene names available in Supplementary Figure S3.

**FIGURE 10:**
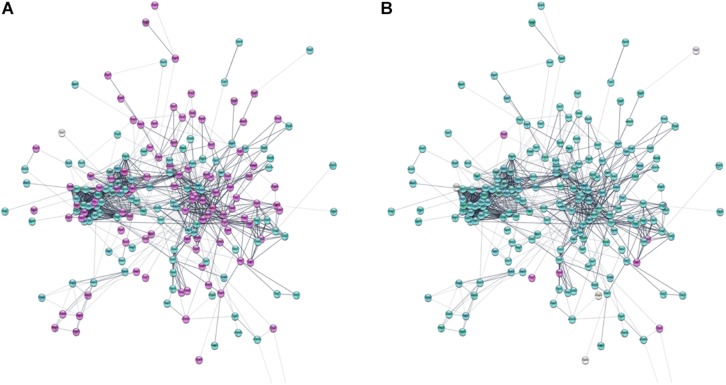
Protein–protein interaction network of differentially expressed genes involved in neuron migration. **(A)** Log2 fold change of gene expression in hNPC/shWWOX vs. hNPC/shScrambled. **(B)** Log2 fold change of gene expression in neurons/shWWOX vs. neurons/shScrambled. Significantly upregulated genes are magenta; significantly downregulated genes are turquoise (Log2 fold change > ±1, *p* < 0.05). High resolution figure with legible gene names available in Supplementary Figures S4, S5.

Based on information from the STRING database, the protein–protein global interaction network was constructed with Log2 fold change of differentially expressed genes belonging to gene sets related to membrane proteins, cytoskeleton and cell signaling. The selected genes encode proteins that play an important role in the steps of CNS development – neurogenesis, neuron migration, and synaptogenesis. The constructed network comprised of 198 nodes and 1113 edges (Figure 9; for details see Supplementary Figure S3). Figure 10 presents protein–protein interaction networks of genes which are differentially expressed between undifferentiated hNPC (A) and differentiated neurons (B). In hNPC, the most significantly upregulated genes (Log2 fold change > ±1, *p* < 0.05) are associated with signaling and chromatin remodeling.

### Principle Component Analysis

**FIGURE 11:**
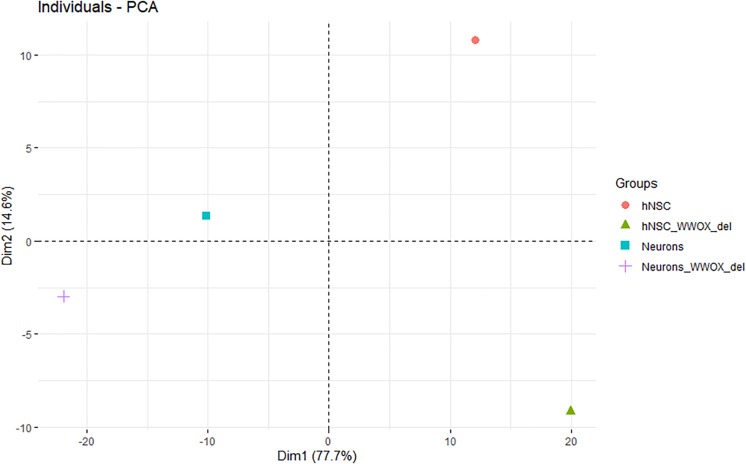
The principle component analysis employing the gene set of oxidative gene set for identification of genes of the highest contribution to spatial partitioning of the genotypes and selection of genes differentiating hNPC/shScrambled, hNPC/shWWOX, neurons/shScrambled and neurons/shWWOX in the most significant manner. The partitioning of hNPC and neuron genotypes across the first dimension. The partitioning of shScrambled control and shWWOX genotypes across the second dimension.

Principle component analysis employing the oxidative gene set served to identify genes of the highest contribution to spatial partitioning of the genotypes and selection of genes differentiating hNPC/shScrambled, hNPC/shWWOX, neurons/shScrambled and neurons/shWWOX in the most significant manner. As expected, we obviously found clear partitioning of hNPC and neuronal genotypes across the first dimension with a total variance of 77.7%. However, we also observed a clear partitioning across the dim2 with a total variance of 14.6%. The dim2 differentiates shScrambled and shWWOX variants indicating changes in oxidation-related genes induced by *WWOX* depletion (Figure 11). PCA analysis showed that among oxidative catabolism and oxidative stress genes that expression differentiates between *WWOX* expressing and *WWOX* depleted hNPC as well as neurons are those important for energy and redox metabolism. Examples of genes mostly involved in such differentiation are: Hexokinase 2 (HK2), Pyruvate Dehydrogenase Kinase 1 (PDK1), Lactate Dehydrogenase A (LDHA), Ferredoxin Reductase (FDXA), Cytochrome C (CYCS), Superoxide Dismutase 1 and 2 (SOD1 and SOD2), and Peroxiredoxin 2 (PRDX2), see Supplementary Table S1 for all involved genes.

## Discussion

The *WWOX* tumor suppressor gene has been recently recognized as a global modulator of transcription and an important regulator of brain differentiation and maintenance. Aberrations in *WWOX* expression lead to severe developmental neuropathologies as well as metabolic insufficiency and premature death (Shaukat et al., 2018). Public data from the Fantom 5 Project of the Riken Database (Robinson et al., 2010; Lizio et al., 2015) indicate that in human brain, *WWOX* expression is strongly dependent on the brain compartment as well as the age of the organism. In our study, we aimed to clarify the molecular basis of *WWOX* actions in neural tissue by analyzing the changes in phenotype and gene expression in hNPC with silenced *WWOX*.

The process of brain development is highly organized and its precise orchestration is vital to ensure the perfect construction of such a complex organ. Neurons and glial cells must be created in accurate numbers, locations and timing. The differentiation of neural stem cells into neurons, as well as the maintenance of the pool of undifferentiated neural progenitors, is governed by intra and extracellular signaling and involves mainly Notch, Wnt, and Hedgehog signaling pathways (Jiang and Nardelli, 2016). Our analysis of the functional networks of the differentially expressed genes revealed that *WWOX* expression in hNPC is associated with transcription regulation of genes of neural differentiation that take part in such pathways as Notch, PI3K kinase, PDGF, Cadherin, Hedgehog, and Endothelin signaling pathway.

The migration of the newborn neurons to their destination sites requires several major changes in cell polarity and motility. Young neurons leaving the SVZ exhibit multipolarity, and those on their way to target site typically display a bipolar morphology (Marín et al., 2006). This elaborate process is guided by intricate intra and extracellular signals. The pivotal role in migration is played by cytoskeleton components such as actin filaments and microtubules, as well as motor proteins like dyneins and kinesins, which undergo strictly controlled rearrangements to facilitate directional movement (Jiang and Nardelli, 2016). We showed that *WWOX* silencing in neural progenitor cells significantly changed expression of a large set of genes connected to cytoskeleton organization, e.g., MAP2/4/6, DCLK, NEFM, and NEFL that are engaged in microtubule and neurofilament assembly. Spalice et al. (2009) showed that impairment in the function of cytoskeletal genes, such as those associated with tubulins, causes severe disturbances in gyrification and lissencephaly, i.e., lack of brain folds and grooves (Liu, 2011). This emphasize the importance of migration process in cortex development.

During synaptogenesis the targets identification by the protruding ends of the outgrowing axons is mediated by cell adhesion molecules, e.g., NCAMs, N-cadherin and integrins, as well as extracellular matrix proteins (Dickson, 2002). Repulsive or attractive cues triggers signalization leading to reorganization of cytoskeleton facilitating directional movement. A number of cell-adhesion proteins is also involved in pre- and postsynaptic specialization (Li and Sheng, 2003). In our study, the lack of *WWOX* expression resulted in significant changes in cellular adhesion to ECM proteins. The hNPC/sh*WWOX* cells demonstrated increased ability to adhere to fibronectin (*p* < 0.05) and to the ECM protein mixture (*p* = 0.0626). Previous studies conducted on various cancer cell lines also identified a negative correlation between *WWOX* expression level and adherence ability (Zhang et al., 2009; Paige et al., 2010; Kośla et al., 2014). Our data indicates that WWOX is an important regulator of the tissue architecture. The changes in *WWOX* expression influence the levels of a number of pivotal adherence proteins, e.g., CDH2 (expression doubled in hNPC/shScrambled in compare to hNPC/sh*WWOX*) and ITGβ1 (expression lowered twice in hNPC/shScrambled in compare to hNPC/sh*WWOX*). The amount of secreted metalloproteinases (pro-MMP2 and pro-MMP9) was also significantly lowered in *WWOX* depleted cells. Metalloproteinases play a major role in the process of migration for neural progenitor cells and neural crest cells (Wang et al., 2006; Monsonego-Ornan et al., 2012). The interaction between ECM proteins and neural stem/progenitor cells is essential for the process of neurodifferentiation and functional maturation (Li et al., 2014). In addition to differentiation, the biochemical and biomechanical interaction with the extracellular microenvironment is also crucial for neural cell migration during the development of the nervous system, as well as in the case of brain injury (Joo et al., 2015). The precise nature of the effect of WWOX on cell adhesion to ECM proteins remain unclear, but aberrations in WWOX protein level or functionality may disturb this physiologically relevant interactions.

The other main change observed in hNPC phenotype concerned mitochondrial redox potential. A number of papers have presented data suggesting that WWOX is capable of modulating cell metabolism (O’Keefe et al., 2011; Dayan et al., 2013; Abu-Remaileh et al., 2014; Choo et al., 2015). In the presented study, hNPC with silenced *WWOX* exhibit significantly lowered mitochondrial redox potential. Reactive oxygen species levels and mitochondrial metabolic state strongly contribute to neurogenesis and neuron maturation. The balance in this processes is critical for maintaining cellular homeostasis and accurate source, timing and localization of ROS synthesis is crucial for neuron physiology (Beckervordersandforth, 2017; Wilson et al., 2018). In our study, the transcriptome of investigated cells was analyzed to look for a confirmation of the observed decrease of the redox potential in *WWOX* depleted hNPC. A PCA with an oxidative stress response/antioxidant defense gene set was employed. The analysis clearly shows that in the terms of the resultant effect of the considered genes’ expression, the control and *WWOX*-depleted genotypes are completely different (Figure 11). This is true both for undifferentiated hNPC and differentiated neurons. Amongst the genes with the highest contribution to differentiation between *WWOX* high/low variants are several important players participating in basic metabolism and reactive oxygen species control (Supplementary Table S1). Therefore, we conclude that *WWOX* might have considerable impact on neurons through modulation of oxygen metabolism and mitochondria functioning.

The specific mechanisms that underlie the individual stages of brain development process are far from being understood. Even so, our findings suggest that the WWOX protein might be an important regulator of neuronal migration and synaptogenesis. Its expression downregulation is significantly associated with changes in the level of expression of the genes involved in cell motility and adhesion, regulation of chromatin condensation and global cell signaling. The gene expression alternations identified in the CAGE analysis are consistent with phenotypical changes including cell adhesion, motility and ability for 3D growth.

The most striking observation in hNPC phenotype was noted when the cells were cultured in a three dimensional scaffold formed of ECM protein mixture. Under these conditions, the hNPC/sh*WWOX* cells lost their potential for 3D growth. They remained as single cells that did neither proliferate nor differentiate, whereas hNPC/shScrambled controls formed extensive cellular networks. This effect of *WWOX* depletion was observed irrespective of seeding density. This observation, along with changes in cellular adhesion, suggests that *WWOX* deprivation may lead to deficiency in cytoskeleton organization, interactions between cells and extracellular matrix proteins that impair correct tissue formation. In fact, human individuals known to possess dysfunctional *WWOX* variants present physical abnormalities in brain structure, *inter alia* global atrophy and hypoplasia of the corpus callosum (Abdel-Salam et al., 2014; Elsaadany et al., 2016; Johannsen et al., 2018). Similarly, Abdeen et al. (2013) report that silencing *WWOX* expression in MCF-10A normal breast cell line caused impairment of 3D growth and increases levels of fibronectin. In our previous report we demonstrated that differentiation of *WWOX* expression influences the 3D growth of the GBM T98G cell line, with upregulation of its expression disturbing the growth and spread of cancer cells in an ECM matrix (Kośla et al., 2014). This inability to perform 3D growth might by implied by massive gene expression deregulation triggered by *WWOX* depletion.

To identify which specific pathways may be influenced by WWOX protein in hNPC, the transcription profiles of undifferentiated hNPC cells and hNPC differentiated into neurons were compared between *WWOX* high and *WWOX* low variants and analyzed ontologically to match the differentially expressed genes with the BP. The global analysis showed enrichment in 14 gene sets related to neuronal development and differentiation (e.g., neuronal progenitor cell population maintenance, regulation of neuron migration, neural crest cell differentiation) (Supplementary Table S2). It was found that the gene sets were enriched in hNPC/shScrambled cells versus hNPC/sh*WWOX*, indicating that WWOX-induced transcription downregulation severely interferes with CNS developmental processes. Our results shows that beside adhesion membrane proteins, *WWOX* expression also influences a significant set of genes engaged in cytoskeleton structure and intracellular signaling. This can directly imply the observed disability for 3D growth and differentiation. *In vivo* the change in cell polarity and morphology is needed to allow migration from neurogenic niches to the final localization, and *WWOX* silencing appears to cause deregulation of the genes crucial for microtubule and neurofilament assembly as well as motor proteins.

The other large functional groups emerging from the ontological analysis were the gene sets connected with cellular adhesion and neurotransmitter management. It was shown recently that in a murine model, *WWOX* knockout may lower GABA synthesis (Hussain et al., 2019).

The multiplicity of molecular changes caused by *WWOX* depletion highlights its importance for the neural differentiation process. Aberration of its expression and/or protein functionality may disturb the delicate balance of this complicated net of connections and consequently lead to severe neuropathological conditions. WWOX has been postulated to act as linker in various protein crosstalk, an important multifunctional node that connects numerous proteins in many pathways, what may nicely explain the versatile effects of its silencing.

## Conclusion

Our findings indicate that appropriate *WWOX* expression is required for hNPC maintenance and differentiation. The silencing of *WWOX* expression results in global transcriptional changes, with the upregulation of some group of genes and simultaneous downregulation of others. Hence, it appears that the WWOX protein may be one of the master expression regulators in neural tissue.

## Data Availability

The datasets generated for this study can be found in NCBI GEO Database, GSE126075.

## Author Contributions

KK planned and performed the experiments, bioinformatic analysis, and wrote the manuscript. EP contributed to the interpretation of the results. ES-B and MN participated in performing the biological experiments. MO performed the statistical and bioinformatic analysis. AB conceived the idea, performed the bioinformatic analysis of CAGE results, and contributed to writing of the manuscript.

## Conflict of Interest Statement

The authors declare that the research was conducted in the absence of any commercial or financial relationships that could be construed as a potential conflict of interest.

## Footnotes

1 http://bioconductor.org/packages/release/bioc/vignettes/CAGEr/inst/doc/CAGEexp.html

2 http://www.string-db.org

3 http://www.cytoscape.org

4 http://software.broadinstitute.org/gsea/msigdb/index.jsp

5 http://fantom.gsc.riken.jp/

## References

[B1] Abdeen S. K. Salah Z. Khawaled S. Aqeilan R. I. (2013). Characterization of WWOX inactivation in murine mammary gland development. *J. Cell. Physiol.* 228 1391–1396. 10.1002/jcp.24310 23254778

[B2] Abdel-Salam G. Thoenes M. Afifi H. H. Körber F. Swan D. Bolz H. J. (2014). The supposed tumor suppressor gene WWOX is mutated in an early lethal microcephaly syndrome with epilepsy, growth retardation and retinal degeneration. *Orphanet J. Rare Dis.* 9:12. 10.1186/1750-1172-9-12 24456803 PMC3918143

[B3] Abu-Odeh M. Bar-Mag T. Huang H. Kim T. Salah Z. Abdeen S. K. (2014). Characterizing WW domain interactions of tumor suppressor WWOX reveals its association with multiprotein networks. *J. Biol. Chem.* 289 8865–8880. 10.1074/jbc.M113.506790 24550385 PMC3979411

[B4] Abu-Remaileh M. Aqeilan R. I. (2014). Tumor suppressor WWOX regulates glucose metabolism via HIF1α modulation. *Cell Death Differ.* 21 1805–1814. 10.1038/cdd.2014.95 25012504 PMC4211377

[B5] Abu-Remaileh M. Seewaldt V. L. Aqeilan R. I. (2014). WWOX loss activates aerobic glycolysis. *Mol. Cell. Oncol.* 2:e965640. 10.4161/23723548.2014.965640 27308416 PMC4904998

[B6] Aqeilan R. I. Donati V. Palamarchuk A. Trapasso F. Kaou M. Pekarsky Y. (2005). WW domain-containing proteins, WWOX and YAP, compete for interaction with ErbB-4 and modulate its transcriptional function. *Cancer Res.* 65 6764–6772. 10.1158/0008-5472.CAN-05-1150 16061658

[B7] Aqeilan R. I. Palamarchuk A. Weigel R. J. Herrero J. J. Pekarsky Y. Croce C. M. (2004a). Physical and functional interactions between the Wwox tumor suppressor protein and the AP-2gamma transcription factor. *Cancer Res.* 64 8256–8261. 10.1158/0008-5472.CAN-04-2055 15548692

[B8] Aqeilan R. I. Pekarsky Y. Herrero J. J. Palamarchuk A. Letofsky J. Druck T. (2004b). Functional association between Wwox tumor suppressor protein and p73, a p53 homolog. *Proc. Natl. Acad. Sci. U.S.A.* 101 4401–4406. 10.1073/pnas.0400805101 15070730 PMC384759

[B9] Beckervordersandforth R. (2017). Mitochondrial metabolism-mediated regulation of adult neurogenesis. *Brain Plast.* 3 73–87. 10.3233/BPL-170044 29765861 PMC5928529

[B10] Bouteille N. Driouch K. Hage P. E. Sin S. Formstecher E. Camonis J. (2009). Inhibition of the Wnt/beta-catenin pathway by the WWOX tumor suppressor protein. *Oncogene* 28 2569–2580. 10.1038/onc.2009.120 19465938

[B11] Chen S.-J. Huang S.-S. Chang N.-S. (2013). Role of WWOX and NF-kappaB in lung cancer progression. *Transl. Respir. Med.* 1:15. 10.1186/2213-0802-1-15 27234396 PMC4715152

[B12] Chen S. T. Chuang J. I. Wang J. P. Tsai M. S. Li H. Chang N.-S. (2004). Expression of WW domain-containing oxidoreductase WOX1 in the developing murine nervous system. *Neuroscience* 124 831–839. 10.1016/j.neuroscience.2003.12.036 15026124

[B13] Chiang M.-F. Chen H.-H. Chi C.-W. Sze C.-I. Hsu M.-L. Shieh H.-R. (2015). Modulation of sonic hedgehog signaling and WW domain containing oxidoreductase WOX1 expression enhances radiosensitivity of human glioblastoma cells. *Exp. Biol. Med.* 240 392–399. 10.1177/1535370214565989 25595187 PMC4935234

[B14] Choo A. O’Keefe L. V. Lee C. S. Gregory S. L. Shaukat Z. Colella A. (2015). Tumor suppressor WWOX moderates the mitochondrial respiratory complex. *Genes Chromosomes Cancer* 54 745–761. 10.1002/gcc.22286 26390919

[B15] Dayan S. O’Keefe L. V. Choo A. Richards R. I. (2013). Common chromosomal fragile site FRA16D tumor suppressor WWOX gene expression and metabolic reprograming in cells. *Genes Chromosomes Cancer* 52 823–831. 10.1002/gcc.22078 23765596

[B16] Dias E. P. Pimenta F. J. Sarquis M. S. Dias Filho M. A. Aldaz C. M. Fujii J. B. (2007). Association between decreased WWOX protein expression and thyroid cancer development. *Thyroid* 17 1055–1059. 10.1089/thy.2007.0232 18047428 PMC4150466

[B17] Dickson B. J. (2002). Molecular mechanisms of axon guidance. *Science* 298 1959–1964. 10.1126/science.1072165 12471249

[B18] Donati V. Fontanini G. Dell’Omodarme M. Prati M. C. Nuti S. Lucchi M. (2007). WWOX expression in different histologic types and subtypes of non-small cell lung cancer. *Clin. Cancer Res.* 13 884–891. 10.1158/1078-0432.CCR-06-2016 17289881

[B19] Elsaadany L. El-Said M. Ali R. Kamel H. Ben-Omran T. (2016). W44X mutation in the WWOX gene causes intractable seizures and developmental delay: a case report. *BMC Med. Genet.* 17:53. 10.1186/s12881-016-0317-z 27495153 PMC4975905

[B20] Ferguson B. W. Gao X. Zelazowski M. J. Lee J. Jeter C. R. Abba M. C. (2013). The cancer gene WWOX behaves as an inhibitor of SMAD3 transcriptional activity via direct binding. *BMC Cancer* 13:593. 10.1186/1471-2407-13-593 24330518 PMC3871008

[B21] Gaudio E. Palamarchuk A. Palumbo T. Trapasso F. Pekarsky Y. Croce C. M. (2006). Physical association with WWOX suppresses c-Jun transcriptional activity. *Cancer Res.* 66 11585–11589. 10.1158/0008-5472.CAN-06-3376 17178850

[B22] Haberle V. Forrest A. R. R. Hayashizaki Y. Carninci P. Lenhard B. (2015). *CAGEr*: precise TSS data retrieval and high-resolution promoterome mining for integrative analyses. *Nucleic Acids Res.* 43:e51. 10.1093/nar/gkv054 25653163 PMC4417143

[B23] Hasegawa A. Daub C. Carninci P. Hayashizaki Y. Lassmann T. (2014). MOIRAI: a compact workflow system for CAGE analysis. *BMC Bioinformatics* 15:144. 10.1186/1471-2105-15-144 24884663 PMC4033680

[B24] Hsu L.-J. Chiang M.-F. Sze C.-I. Su W.-P. Yap Y. V. Lee I.-T. (2016). HYAL-2-WWOX-SMAD4 signaling in cell death and anticancer response. *Front. Cell Dev. Biol.* 4:141. 10.3389/fcell.2016.00141 27999774 PMC5138198

[B25] Hussain T. Kil H. Hattiangady B. Lee J. Kodali M. Shuai B. (2019). Wwox deletion leads to reduced GABA-ergic inhibitory interneuron numbers and activation of microglia and astrocytes in mouse hippocampus. *Neurobiol. Dis.* 121 163–176. 10.1016/j.nbd.2018.09.026 30290271 PMC7104842

[B26] Iatan I. Choi H. Y. Ruel I. Reddy M. V. P. L. Kil H. Lee J. (2014). The WWOX gene modulates high-density lipoprotein and lipid metabolism. *Circ. Cardiovasc. Genet.* 7 491–504. 10.1161/CIRCGENETICS.113.000248 24871327 PMC4315188

[B27] Jiang X. Nardelli J. (2016). Cellular and molecular introduction to brain development. *Neurobiol. Dis.* 92 3–17. 10.1016/j.nbd.2015.07.007 26184894 PMC4720585

[B28] Johannsen J. Kortüm F. Rosenberger G. Bokelmann K. Schirmer M. A. Denecke J. (2018). A novel missense variant in the SDR domain of the WWOX gene leads to complete loss of WWOX protein with early-onset epileptic encephalopathy and severe developmental delay. *Neurogenetics* 19 151–156. 10.1007/s10048-018-0549-545 29808465

[B29] Joo S. Kim J. Y. Lee E. Hong N. Sun W. Nam Y. (2015). Effects of ECM protein micropatterns on the migration and differentiation of adult neural stem cells. *Sci. Rep.* 5:13043. 10.1038/srep13043 26266893 PMC4533601

[B30] Kośla K. Nowakowska M. Pospiech K. Bednarek A. K. (2014). WWOX modulates the gene expression profile in the T98G glioblastoma cell line rendering its phenotype less malignant. *Oncol. Rep.* 32 1362–1368. 10.3892/or.2014.3335 25051421 PMC4148378

[B31] Kosla K. Pluciennik E. Kurzyk A. Jesionek-Kupnicka D. Kordek R. Potemski P. (2011). Molecular analysis of WWOX expression correlation with proliferation and apoptosis in glioblastoma multiforme. *J. Neurooncol.* 101 207–213. 10.1007/s11060-010-0254-251 20535528 PMC2996532

[B32] Kuhn R. M. Haussler D. Kent W. J. (2013). The UCSC genome browser and associated tools. *Brief. Bioinform.* 14 144–161. 10.1093/bib/bbs038 22908213 PMC3603215

[B33] Lan C. Chenggang W. Yulan B. Xiaohui D. Junhui Z. Xiao W. (2012). Aberrant expression of WWOX protein in epithelial ovarian cancer: a clinicopathologic and immunohistochemical study. *Int. J. Gynecol. Pathol.* 31 125–132. 10.1097/PGP.0b013e3182297fd2 22317867

[B34] Li Y. Liu M. Yan Y. Yang S.-T. (2014). Neural differentiation from pluripotent stem cells: the role of natural and synthetic extracellular matrix. *World J. Stem Cells* 6 11–23. 10.4252/wjsc.v6.i1.11 24567784 PMC3927010

[B35] Li Z. Sheng M. (2003). Some assembly required: the development of neuronal synapses. *Nat. Rev. Mol. Cell Biol.* 4 833–841. 10.1038/nrm1242 14625534

[B36] Liu C.-C. Ho P.-C. Lee I.-T. Chen Y.-A. Chu C.-H. Teng C.-C. (2018). WWOX phosphorylation, signaling, and role in neurodegeneration. *Front. Neurosci.* 12:563. 10.3389/fnins.2018.00563 30158849 PMC6104168

[B37] Liu J. S. (2011). Molecular genetics of neuronal migration disorders. *Curr. Neurol. Neurosci. Rep.* 11 171–178. 10.1007/s11910-010-0176-175 21222180

[B38] Lizio M. Harshbarger J. Shimoji H. Severin J. Kasukawa T. Sahin S. (2015). Gateways to the FANTOM5 promoter level mammalian expression atlas. *Genome Biol.* 16:22. 10.1186/s13059-014-0560-566 25723102 PMC4310165

[B39] Lo C.-P. Hsu L.-J. Li M.-Y. Hsu S.-Y. Chuang J.-I. Tsai M.-S. (2008). MPP+-induced neuronal death in rats involves tyrosine 33 phosphorylation of WW domain-containing oxidoreductase WOX1. *Eur. J. Neurosci.* 27 1634–1646. 10.1111/j.1460-9568.2008.06139.x 18371080

[B40] Mallaret M. Synofzik M. Lee J. Sagum C. A. Mahajnah M. Sharkia R. (2014). The tumour suppressor gene WWOX is mutated in autosomal recessive cerebellar ataxia with epilepsy and mental retardation. *Brain J. Neurol.* 137 411–419. 10.1093/brain/awt338 24369382 PMC3914474

[B41] Marín O. Valdeolmillos M. Moya F. (2006). Neurons in motion: same principles for different shapes? *Trends Neurosci.* 29 655–661. 10.1016/j.tins.2006.10.001 17046074

[B42] Matteucci E. Bendinelli P. Desiderio M. A. (2009). Nuclear localization of active HGF receptor met in aggressive MDA-MB231 breast carcinoma cells. *Carcinogenesis* 30 937–945. 10.1093/carcin/bgp080 19357348

[B43] Mignot C. Lambert L. Pasquier L. Bienvenu T. Delahaye-Duriez A. Keren B. (2015). WWOX-related encephalopathies: delineation of the phenotypical spectrum and emerging genotype-phenotype correlation. *J. Med. Genet.* 52 61–70. 10.1136/jmedgenet-2014-102748 25411445

[B44] Monsonego-Ornan E. Kosonovsky J. Bar A. Roth L. Fraggi-Rankis V. Simsa S. (2012). Matrix metalloproteinase 9/gelatinase B is required for neural crest cell migration. *Dev. Biol.* 364 162–177. 10.1016/j.ydbio.2012.01.028 22342386

[B45] Nunez M. I. Ludes-Meyers J. Aldaz C. M. (2006). WWOX protein expression in normal human tissues. *J. Mol. Histol.* 37 115–125. 10.1007/s10735-006-9046-9045 16941225 PMC4144810

[B46] O’Keefe L. V. Colella A. Dayan S. Chen Q. Choo A. Jacob R. (2011). Drosophila orthologue of WWOX, the chromosomal fragile site FRA16D tumour suppressor gene, functions in aerobic metabolism and regulates reactive oxygen species. *Hum. Mol. Genet.* 20 497–509. 10.1093/hmg/ddq495 21075834 PMC3016910

[B47] Pagés H. (2018). *BSgenome: Software Infrastructure for Efficient Representation of Full Genomes and Their SNPs. R Package Version 1.48.0.* Available at: http://bioconductor.riken.jp/packages/3.7/bioc/html/BSgenome.html

[B48] Paige A. J. W. Zucknick M. Janczar S. Paul J. Mein C. A. Taylor K. J. (2010). WWOX tumour suppressor gene polymorphisms and ovarian cancer pathology and prognosis. *Eur. J. Cancer* 46 818–825. 10.1016/j.ejca.2009.12.021 20074932

[B49] Piard J. Hawkes L. Milh M. Villard L. Borgatti R. Romaniello R. (2018). The phenotypic spectrum of WWOX-related disorders: 20 additionalcases of WOREE syndrome and review of the literature. *Genet. Med.* 21 1308–1318. 10.1038/s41436-018-0339-3 30356099 PMC6752669

[B50] Płuciennik E. Kusińska R. Potemski P. Kubiak R. Kordek R. Bednarek A. K. (2006). WWOX–the FRA16D cancer gene: expression correlation with breast cancer progression and prognosis. *Eur. J. Surg. Oncol.* 32 153–157. 10.1016/j.ejso.2005.11.002 16360296

[B51] Płuciennik E. Nowakowska M. Stȩpien A. Wołkowicz M. Stawiński A. Różański W. (2014). Alternating expression levels of WWOX tumor suppressor and cancer-related genes in patients with bladder cancer. *Oncol. Lett.* 8 2291–2297. 10.3892/ol.2014.2476 25295115 PMC4186597

[B52] Robinson M. D. McCarthy D. J. Smyth G. K. (2010). edgeR: a bioconductor package for differential expression analysis of digital gene expression data. *Bioinformatics* 26 139–140. 10.1093/bioinformatics/btp616 19910308 PMC2796818

[B53] Seabra M. A. L. Cândido E. B. Vidigal P. V. T. Lamaita R. M. Rodrigues A. N. Silva Filho A. L. D. (2018). Immunohistochemical WWOX expression and association with angiogenesis, p53 expression, cell proliferation and clinicopathological parameters in cervical cancer. *Rev. Bras. Ginecol. Obstet.* 40 79–85. 10.1055/s-0037-1618597 29310145 PMC10309299

[B54] Shaukat Q. Hertecant J. El-Hattab A. W. Ali B. R. Suleiman J. (2018). West syndrome, developmental and epileptic encephalopathy, and severe CNS disorder associated with WWOX mutations. *Epileptic Disord.* 20 401–412. 10.1684/epd.2018.1005 30361190

[B55] Spalice A. Parisi P. Nicita F. Pizzardi G. Del Balzo F. Iannetti P. (2009). Neuronal migration disorders: clinical, neuroradiologic and genetics aspects. *Acta Paediatr.* 98 421–433. 10.1111/j.1651-2227.2008.01160.x 19120042

[B56] Sun W. Dou J. Zhang L. Qiao L. Shen N. Gao W. (2017). Expression of CD133, E-cadherin and WWOX in colorectal cancer and related analysis. *Pak. J. Med. Sci.* 33 425–429. 10.12669/pjms.332.11687 28523049 PMC5432716

[B57] Suzuki H. Katayama K. Takenaka M. Amakasu K. Saito K. Suzuki K. (2009). A spontaneous mutation of the Wwox gene and audiogenic seizures in rats with lethal dwarfism and epilepsy. *Genes Brain Behav.* 8 650–660. 10.1111/j.1601-183X.2009.00502.x 19500159

[B58] Sze C.-I. Su M. Pugazhenthi S. Jambal P. Hsu L.-J. Heath J. (2004). Down-regulation of WW domain-containing oxidoreductase induces tau phosphorylation in vitro. A potential role in Alzheimer’s disease. *J. Biol. Chem.* 279 30498–30506. 10.1074/jbc.M401399200 15126504

[B59] Tabarki B. Al Mutairi F. Al Hashem A. (2015a). The fragile site WWOX gene and the developing brain. *Exp. Biol. Med.* 240 400–402. 10.1177/1535370214561952 25416187 PMC4935222

[B60] Tabarki B. AlHashem A. AlShahwan S. Alkuraya F. S. Gedela S. Zuccoli G. (2015b). Severe CNS involvement in WWOX mutations: description of five new cases. *Am. J. Med. Genet. A.* 167 3209–3213. 10.1002/ajmg.a.37363 26345274

[B61] Takahashi H. Lassmann T. Murata M. Carninci P. (2012). 5’ end-centered expression profiling using cap-analysis gene expression and next-generation sequencing. *Nat. Protoc.* 7 542–561. 10.1038/nprot.2012.005 22362160 PMC4094379

[B62] Teng C.-C. Yang Y.-T. Chen Y.-C. Kuo Y.-M. Sze C.-I. (2012). Role of WWOX/WOX1 in Alzheimer’s disease pathology and in cell death signaling. *Front. Biosci.* 4:1951–1965. 10.2741/e516 22202011

[B63] Wang H.-Y. Juo L.-I. Lin Y.-T. Hsiao M. Lin J.-T. Tsai C.-H. (2012). WW domain-containing oxidoreductase promotes neuronal differentiation via negative regulation of glycogen synthase kinase 3β. *Cell Death Differ.* 19 1049–1059. 10.1038/cdd.2011.188 22193544 PMC3354054

[B64] Wang L. Zhang Z. G. Zhang R. L. Gregg S. R. Hozeska-Solgot A. LeTourneau Y. (2006). Matrix metalloproteinase 2 (MMP2) and MMP9 secreted by erythropoietin-activated endothelial cells promote neural progenitor cell migration. *J. Neurosci.* 26 5996–6003. 10.1523/JNEUROSCI.5380-05.2006 16738242 PMC6675216

[B65] Wang X. Chao L. Ma G. Chen L. Zang Y. Sun J. (2011). The prognostic significance of WWOX expression in patients with breast cancer and its association with the basal-like phenotype. *J. Cancer Res. Clin. Oncol.* 137 271–278. 10.1007/s00432-010-0880-881 20401669 PMC11828298

[B66] Wilson C. Muñoz-Palma E. González-Billault C. (2018). From birth to death: a role for reactive oxygen species in neuronal development. *Semin. Cell Dev. Biol.* 80 43–49. 10.1016/j.semcdb.2017.09.012 28899716

[B67] Xiong A. Wei L. Ying M. Wu H. Hua J. Wang Y. (2014). Wwox suppresses breast cancer cell growth through modulation of the hedgehog-GLI1 signaling pathway. *Biochem. Biophys. Res. Commun.* 443 1200–1205. 10.1016/j.bbrc.2013.12.133 24393846

[B68] Zhang J. Li L. Song H. Paige A. Gabra H. (2009). [Effect of WWOX gene on the attachment and adhesion of ovarian cancer cells]. *Zhonghua Fu Chan Ke Za Zhi* 44 529–532. 19957554

