# Supplementary material for: The WWOX Gene Influences Cellular Pathways in the Neuronal Differentiation of Human Neural Progenitor Cells
Source: Front Cell Neurosci. 2019 Aug 30;13:391. doi: 10.3389/fncel.2019.00391 (PMC6730490; doi:10.3389/fncel.2019.00391)
Supplement: Supplementary file 1 [file Data_Sheet_1.pdf]

Supplementary Material

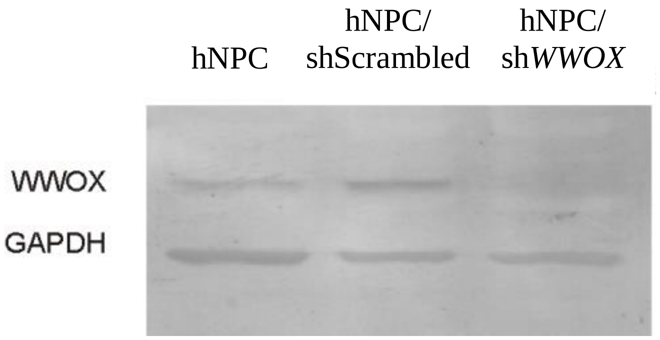

Supplementary Figure 1. Western Blot verification of *WWOX* gene silencing. From left: native hNPC, hNPC/shScrambled control and hNPC/*shWWOX* silenced variant.

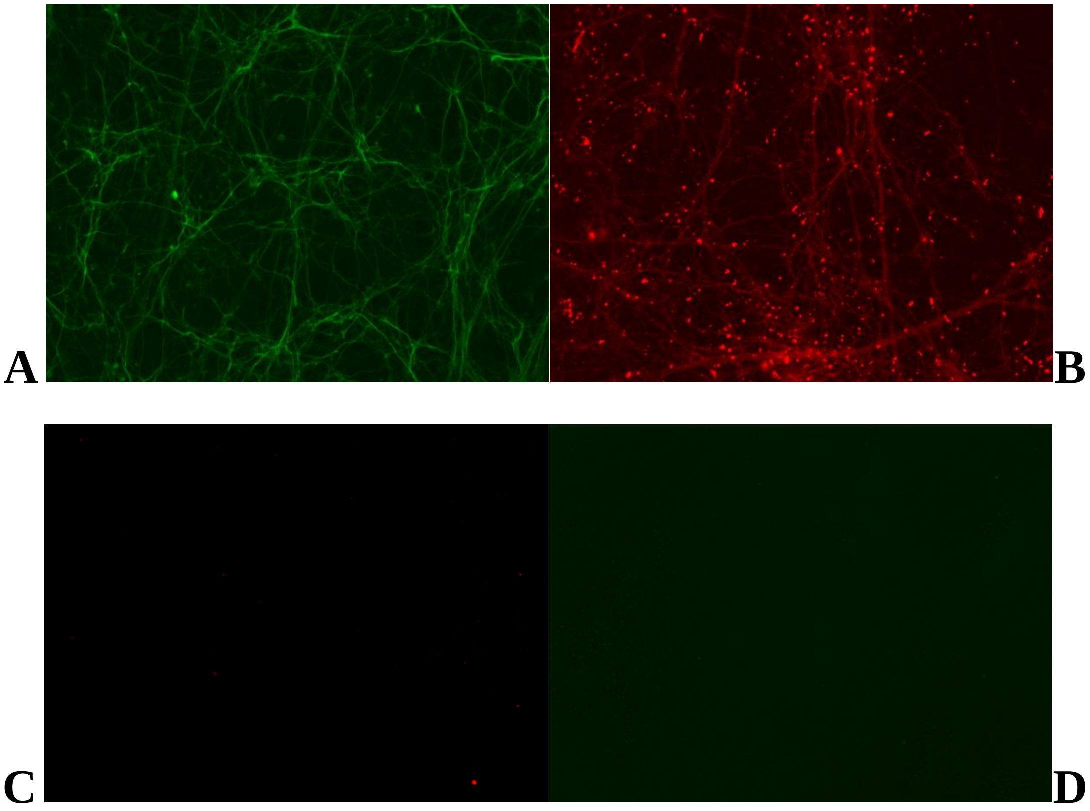

Supplementary Figure 2. Immunocytochemistry staining of spontaneously differentiated human neural progenitor cells (H9 derived) with cell-type specific markers. Neuronal markers MAP2 and TUJ (A, B), astrocyte marker GFAP (C) and oligodendrocyte marker GalC (D).

Supplementary Figures 3-5 available as separate **pdf** files, for the reason of maintaining high quality graphics.

Supplementary Table 1. The selected genes with high contribution to spatial partitioning of the hNPC/shScrambled, hNPC/shWWOX, neurons/shScrambled and neurons/shWWOX genotypes in the principal component analysis (PCA) with an oxidative stress response/antioxidant defense gene set. Protein function descriptions according to GeneCards®: The Human Gene Database.

Gene	Contribution	Protein name	Function of encoded protein
SPR	1.176	Sepiapterin Reductase	This gene encodes an aldo-keto reductase that catalyzes the NADPH-dependent reduction of pteridine derivatives and is important in the biosynthesis of tetrahydrobiopterin (BH4). Among its related pathways are eNOS activation and regulation and Folate biosynthesis. Gene Ontology (GO) annotations related to this gene include oxidoreductase activity and aldo-keto reductase (NADP) activity.
PXDN	0.976	Peroxidasin	Displays low peroxidase activity and is likely to participate in H_2O_2 metabolism and peroxidative reactions in the cardiovascular system. Plays a role in extracellular matrix formation.
ADCY3	0.914	Adenylate Cyclase 3	Catalyzes the formation of the signaling molecule cAMP in response to G-protein signaling. Participates in signaling cascades triggered by odorant receptors via its function in cAMP biosynthesis.
NFE2L2	0.821	Nuclear Factor, Erythroid 2 Like 2	Transcription activator that binds to antioxidant response (ARE) elements in the promoter regions of target genes. Important for the coordinated up-regulation of genes in response to oxidative stress and the regulation of cellular redox conditions.
CHD6	0.782	Chromodomain Helicase DNA Binding Protein 6	DNA-dependent ATPase that plays a role in chromatin remodeling. Activates transcription of specific genes in response to oxidative stress through interaction with NFE2L2.
SRXN1	0.781	Sulfiredoxin 1	Contributes to oxidative stress resistance by reducing cysteine-sulfinic acid formed under exposure to oxidants in the peroxiredoxins PRDX1, PRDX2, PRDX3 and PRDX4. Does not act on PRDX5 or PRDX6. May catalyze the reduction in a multi-step process by acting both as a specific phosphotransferase and a thioltransferase.
PDK1	0.726	Pyruvate Dehydrogenase Kinase 1	Pyruvate dehydrogenase (PDH) is a mitochondrial multienzyme complex that catalyzes the oxidative decarboxylation of pyruvate and is one of the major enzymes responsible for the regulation of homeostasis of carbohydrate fuels in mammals. The enzymatic activity is regulated by a phosphorylation/dephosphorylation cycle. Phosphorylation of PDH by a specific pyruvate dehydrogenase kinase (PDK) results in inactivation.
SEPX1	0.691	Methionine Sulfoxide Reductase B1	Members of this family function as repair enzymes that protect proteins from oxidative stress by catalyzing the reduction of methionine-R-sulfoxides to methionines.
G6PD	0.677	Glucose-6-Phosphate Dehydrogenase	This protein is a cytosolic enzyme encoded by a housekeeping X-linked gene whose main function is to produce NADPH, a key electron donor in the defense against oxidizing agents and in reductive biosynthetic reactions.
CYCS	0.664	Cytochrome C, Somatic	This gene encodes a small heme protein that functions as a central component of the electron transport chain in mitochondria. The encoded protein associates with the inner membrane of the mitochondrion where it accepts electrons from cytochrome b and transfers them to the cytochrome oxidase complex.

UCP2	0.658	Uncoupling Protein 2	Reduce the mitochondrial membrane potential in mammalian cells. UCP are mitochondrial transporter proteins that create proton leaks across the inner mitochondrial membrane, thus uncoupling oxidative phosphorylation from ATP synthesis. As a result, energy is dissipated in the form of heat.
TXNIP	0.633	Thioredoxin Interacting Protein	This protein inhibits the antioxidative function of thioredoxin resulting in the accumulation of reactive oxygen species and cellular stress. This protein also functions as a regulator of cellular metabolism and of endoplasmic reticulum (ER) stress. May act as an oxidative stress mediator by inhibiting thioredoxin activity or by limiting its bioavailability.
FXN	0.631	Frataxin	The protein functions in regulating mitochondrial iron transport and respiration. May play a role in the protection against iron-catalyzed oxidative stress through its ability to catalyze the oxidation of Fe(2+) to Fe(3+);
ARG2	0.599	Arginase 2	May play a role in the regulation of extra-urea cycle arginine metabolism and also in down-regulation of nitric oxide synthesis.
GCLM	0.589	Glutamate-Cysteine Ligase Regulatory Protein	The first rate limiting enzyme of glutathione synthesis.
ALDH3A2	0.574	Aldehyde Dehydrogenase 3 Family Member A2	Catalyzes the oxidation of long-chain aliphatic aldehydes to fatty acids.
MSRB3	0.561	Methionine Sulfoxide Reductase B3	The protein encoded by this gene catalyzes the reduction of methionine sulfoxide to methionine.
NDUFS4	0.555	Mitochondrial Respiratory Chain Complex I	Accessory subunit of the mitochondrial membrane respiratory chain NADH dehydrogenase (Complex I), that is believed not to be involved in catalysis. Complex I functions in the transfer of electrons from NADH to the respiratory chain.
FDXR	0.543	Ferredoxin Reductase	This gene encodes a mitochondrial flavoprotein that initiates electron transport for cytochromes P450 receiving electrons from NADPH.
LDHA	0.498	Lactate Dehydrogenase A	It catalyzes the conversion of pyruvate to lactate under anaerobic conditions and is key in the altered glycolytic metabolism that is a feature of cancer cells.
CYBA	0.468	Cytochrome B-245 Alpha Chain	Cytochrome b is comprised of a light chain (alpha) and a heavy chain (beta). This gene encodes the light, alpha subunit which has been proposed as a primary component of the microbicidal oxidase system of phagocytes. Critical component of the membrane-bound oxidase of phagocytes that generates superoxide. Associates with NOX3 to form a functional NADPH oxidase constitutively generating superoxide.
GSK3B	0.435	Glycogen Synthase Kinase 3 Beta	The protein encoded by this gene is a serine-threonine kinase belonging to the glycogen synthase kinase subfamily. It is a negative regulator of glucose homeostasis and is involved in energy metabolism, inflammation, ER-stress, mitochondrial dysfunction, and apoptotic pathways.
DDAH1	0.398	Dimethylarginine Dimethylaminohydrolase 1	Hydrolyzes N(G),N(G)-dimethyl-L-arginine (ADMA) and N(G)-monomethyl-L-arginine (MMA) which act as inhibitors of NOS. Has therefore a role in the regulation of nitric oxide generation.

DDAH2	0.370	Dimethylarginine Dimethylaminohydrolase 2	The encoded enzyme functions in nitric oxide generation by regulating the cellular concentrations of methylarginines, which in turn inhibit nitric oxide synthase activity. The protein may be localized to the mitochondria.
CYB5R4	0.307	Cytochrome B5 Reductase 4	NADH-cytochrome b5 reductase involved in endoplasmic reticulum stress response pathway. Plays a critical role in protecting pancreatic beta-cells against oxidant stress, possibly by protecting the cell from excess buildup of reactive oxygen species (ROS). Reduces a variety of substrates in vitro, such as cytochrome c, feericyanide and methemoglobin.
GUCY1B3	0.252	Guanylate Cyclase 1 Soluble Subunit Beta 1	Mediates responses to nitric oxide (NO) by catalyzing the biosynthesis of the signaling molecule cGMP.
SOD2	0.239	Superoxide Dismutase 2	Destroys superoxide anion radicals which are normally produced within the cells and which are toxic to biological systems.
PFKP	0.200	Phosphofructokinase, Platelet	Catalyzes the phosphorylation of D-fructose 6-phosphate to fructose 1,6-bisphosphate by ATP, the first committing step of glycolysis
HK2	0.144	Hexokinase 2	Hexokinases phosphorylate glucose to produce glucose-6-phosphate, the first step in most glucose metabolism pathways. This gene encodes hexokinase 2, the predominant form found in skeletal muscle. It localizes to the outer membrane of mitochondria.
SLC25A24	0.127	Solute Carrier Family 25 Member 24	Calcium-dependent mitochondrial solute carrier. Mediates the reversible, electroneutral exchange of Mg-ATP or Mg-ADP against phosphate ions, catalyzing the net uptake or efflux of adenine nucleotides across the mitochondrial inner membrane. May play a role in protecting cells against oxidative stress-induced cell death, probably by promoting the formation of calcium-phosphate precipitates in the mitochondrial matrix, and thereby buffering calcium levels in the mitochondrial matrix.
ROMO1	0.120	Reactive Oxygen Species Modulator 1	Induces production of reactive oxygen species (ROS) which are necessary for cell proliferation. May play a role in inducing oxidative DNA damage and replicative senescence. May play a role in the coordination of mitochondrial morphology and cell proliferation.
IDH1	0.095	Isocitrate Dehydrogenase (NADP(+)) 1, Cytosolic	Isocitrate dehydrogenases catalyze the oxidative decarboxylation of isocitrate to 2-oxoglutarate. he cytoplasmic enzyme serves a significant role in cytoplasmic NADPH production.
SOD1	0.092	Superoxide Dismutase 1	Destroys radicals which are normally produced within the cells and which are toxic to biological systems.
PRDX2	0.084	Peroxiredoxin 2	Thiol-specific peroxidase that catalyzes the reduction of hydrogen peroxide and organic hydroperoxides to water and alcohols, respectively. Plays a role in cell protection against oxidative stress by detoxifying peroxides and as sensor of hydrogen peroxide-mediated signaling events.
HIF1A	0.033	Hypoxia Inducible Factor 1 Subunit Alpha	HIF-1 functions as a master regulator of cellular and systemic homeostatic response to hypoxia by activating transcription of many genes, including those involved in energy metabolism, angiogenesis, apoptosis, and other genes whose protein products increase oxygen delivery or facilitate metabolic adaptation to hypoxia.

Supplementary Table 2 GSEA report. Enrichment in phenotype hNPC_WWOX_high.

Gene set name	SIZE	Enrichment score	FDR q-val
GO_NEURAL_CREST_CELL_DIFFERENTIATION	33	0.622	0.000
GO_NEURAL_CREST_CELL_MIGRATION	21	0.677	0.003
GO_HOMOPHILIC_CELL_ADHESION_VIA_PLASMA_MEMBRANE_ADHESION_MOLECULES	53	0.511	0.007
GO_CELL_CELL_ADHESION_VIA_PLASMA_MEMBRANE_ADHESION_MOLECULES	69	0.428	0.101
GO_NEGATIVE_REGULATION_OF_AXON_EXTENSION	21	0.578	0.089
GO_AORTA_DEVELOPMENT	24	0.547	0.078
GO_PHOTORECEPTOR_CELL_MAINTENANCE	15	0.607	0.080
GO_DENDRITE_MORPHOGENESIS	30	0.525	0.072
GO_EMBRYONIC_PATTERN_SPECIFICATION	24	0.533	0.065
GO_REGULATION_OF_NEUROTRANSMITTER_TRANSPORT	27	0.518	0.086
GO_PROTEIN_LIPID_COMPLEX_SUBUNIT_ORGANIZATION	16	0.592	0.099
GO_REGULATION_OF_NEUROTRANSMITTER_SECRETION	23	0.521	0.115
GO_SEGMENTATION	46	0.425	0.111
GO_TISSUE_REMODELING	38	0.449	0.115
GO_REGULATION_OF_NEURON_MIGRATION	17	0.555	0.114
GO_RETINA_HOMEOSTASIS	23	0.503	0.160
GO_NEURON_RECOGNITION	20	0.507	0.183
GO_POSITIVE_REGULATION_OF_LIPID_TRANSPORT	15	0.555	0.190
GO_STEM_CELL_DIFFERENTIATION	80	0.360	0.203
GO_MESENCHYMAL_CELL_DIFFERENTIATION	60	0.391	0.198
GO_REGULATION_OF_PLASMA_LIPOPROTEIN_PARTICLE_LEVELS	21	0.494	0.213
GO_ENDOCHONDRAL_BONE_MORPHOGENESIS	18	0.498	0.221
GO_REGULATION_OF_CARTILAGE_DEVELOPMENT	24	0.471	0.213
GO_NEGATIVE_REGULATION_OF_NEURON_DIFFERENTIATION	98	0.344	0.212
GO_RESPIRATORY_GASEOUS_EXCHANGE	23	0.460	0.204
GO_COLUMNAR_CUBOIDAL_EPITHELIAL_CELL_DEVELOPMENT	19	0.486	0.208
GO_CARDIAC_SEPTUM_DEVELOPMENT	52	0.376	0.218
GO_NEGATIVE_CHEMOTAXIS	22	0.464	0.231
GO_SOMITOGENESIS	34	0.422	0.227
GO_ARTERY_MORPHOGENESIS	22	0.476	0.229
GO_REGULATION_OF_PHOSPHOLIPASE_ACTIVITY	18	0.497	0.222
GO_NEGATIVE_REGULATION_OF_STEM_CELL_DIFFERENTIATION	22	0.470	0.224
GO_NEGATIVE_REGULATION_OF_NERVOUS_SYSTEM_DEVELOPMENT	143	0.305	0.222
GO_MESENCHYME_DEVELOPMENT	82	0.347	0.216
GO_NEGATIVE_REGULATION_OF_WOUND_HEALING	17	0.486	0.223
GO_REGULATION_OF_AXON_GUIDANCE	18	0.480	0.220
GO_REGULATION_OF_CHONDROCYTE_DIFFERENTIATION	21	0.470	0.223
GO_REGULATION_OF_EXTENT_OF_CELL_GROWTH	59	0.369	0.228
GO_REGULATION_OF_NEURAL_PRECURSOR_CELL_PROLIFERATION	36	0.396	0.236
GO_ARTERY_DEVELOPMENT	38	0.389	0.242
GO_NEURONAL_STEM_CELL_POPULATION_MAINTENANCE	16	0.495	0.238
GO_SEMAPHORIN_PLEXIN_SIGNALING_PATHWAY	22	0.458	0.233
GO_DENDRITE_DEVELOPMENT	53	0.366	0.231
GO_REGULATION_OF_AXONOGENESIS	92	0.322	0.234

Supplementary Table 3 GSEA report. Enrichment in phenotype hNPC_WWOX_del.

Gene set name	Size	Enrichment Score	FDR q-val
GO_RESPONSE_TO_ZINC_ION	20	-0.703	0.029
GO_MATURATION_OF_SSU_RRNA	37	-0.603	0.051
GO_NEGATIVE_REGULATION_OF_MYELOID_LEUKOCYTE_DIFFERENTIATION	17	-0.686	0.079
GO_RRNA_TRANSCRIPTION	15	-0.698	0.080
GO_TRNA_METABOLIC_PROCESS	137	-0.463	0.068
GO_ALPHA_AMINO_ACID_BIOSYNTHETIC_PROCESS	48	-0.549	0.065
GO_MATURATION_OF_SSU_RRNA_FROM_TRICISTRONIC_RRNA_TRANSCRIPT_SSU_RRNA_5_8S_RRNA_LSU_RRNA_	30	-0.593	0.057
GO_RIBOSOME_BIOGENESIS	266	-0.429	0.055
GO_NUCLEAR_EXPORT	127	-0.459	0.075
GO_TETRAPYRROLE_METABOLIC_PROCESS	35	-0.556	0.093
GO_ANTIGEN_PROCESSING_AND_PRESENTATION_OF_EXOGENOUS_PEPTIDE_ANTIGEN_VIA_MHC_CLASS_I	49	-0.513	0.093
GO_NEGATIVE_REGULATION_OF_MYELOID_CELL_DIFFERENTIATION	34	-0.559	0.093
GO_ACUTE_INFLAMMATORY_RESPONSE	19	-0.628	0.099
GO_WATER_SOLUBLE_VITAMIN_METABOLIC_PROCESS	48	-0.511	0.103
GO_RIBOSOMAL_SMALL_SUBUNIT_BIOGENESIS	52	-0.500	0.102
GO_OLIGOSACCHARIDE_LIPID_INTERMEDIATE_BIOSYNTHETIC_PROCESS	18	-0.630	0.096
GO_REGULATION_OF_ERAD_PATHWAY	22	-0.597	0.091
GO_INTRASPECIES_INTERACTION_BETWEEN_ORGANISMS	19	-0.627	0.095
GO_SERINE_FAMILY_AMINO_ACID_METABOLIC_PROCESS	26	-0.576	0.098
GO_ANTIGEN_PROCESSING_AND_PRESENTATION_OF_PEPTIDE_ANTIGEN_VIA_MHC_CLASS_I	63	-0.483	0.103
GO_NCRNA_METABOLIC_PROCESS	430	-0.395	0.116
GO_RIBONUCLEOPROTEIN_COMPLEX_BIOGENESIS	373	-0.398	0.115
GO_MESENCHYME_MORPHOGENESIS	18	-0.615	0.112
GO_RIBONUCLEOPROTEIN_COMPLEX_LOCALIZATION	104	-0.440	0.108
GO_TRNA_PROCESSING	85	-0.457	0.113
GO_REGULATION_OF_RECEPTOR_INTERNALIZATION	18	-0.614	0.112
GO_REGULATION_OF_INTERFERON_BETA_PRODUCTION	22	-0.582	0.110
GO_MITOCHONDRIAL_RNA_METABOLIC_PROCESS	22	-0.586	0.110
GO_REGULATION_OF_CELLULAR_RESPONSE_TO_HEAT	58	-0.476	0.111
GO_MITOCHONDRIAL_TRANSLATION	98	-0.437	0.119
GO_ZINC_II_ION_TRANSPORT	16	-0.624	0.134
GO_RRNA_METABOLIC_PROCESS	222	-0.398	0.165
GO_NCRNA_PROCESSING	324	-0.387	0.161
GO_NEGATIVE_REGULATION_OF_ERK1_AND_ERK2_CASCADE	28	-0.536	0.169
GO_TRNA_TRANSPORT	31	-0.522	0.177
GO_NEGATIVE_REGULATION_OF_LEUKOCYTE_DIFFERENTIATION	31	-0.521	0.178
GO_NEGATIVE_REGULATION_OF_DNA_REPLICATION	39	-0.499	0.177
GO_AMINO_ACID_ACTIVATION	43	-0.493	0.173
GO_NEGATIVE_REGULATION_OF_CELL_SUBSTRATE_ADHESION	27	-0.534	0.172
GO_AMINO_ACID_TRANSMEMBRANE_TRANSPORT	21	-0.569	0.175
GO_NUCLEOBASE_CONTAINING_COMPOUND_TRANSPORT	153	-0.406	0.174
GO_NEGATIVE_REGULATION_OF_MITOCHONDRION_ORGANIZATION	26	-0.544	0.171
GO_RNA_MODIFICATION	79	-0.443	0.183
GO_INACTIVATION_OF_MAPK_ACTIVITY	15	-0.624	0.180
GO_HEME_METABOLIC_PROCESS	21	-0.559	0.186
GO_PEPTIDYL_ASPARAGINE_MODIFICATION	28	-0.527	0.188
GO_PROTEIN_EXPORT_FROM_NUCLEUS	24	-0.541	0.191

GO_ER_ASSOCIATED_UBIQUITIN_DEPENDENT_PROTEIN_CATABOLIC_PROCESS	55	-0.456	0.187
GO_TRNA_MODIFICATION	43	-0.489	0.185
GO_GLUCOSE_6_PHOSPHATE_METABOLIC_PROCESS	16	-0.599	0.181
GO_RESPONSE_TO_ANTIBIOTIC	24	-0.543	0.186
GO_TRNA_METHYLATION	19	-0.585	0.183
GO_POSITIVE_REGULATION_OF_INTERFERON_BETA_PRODUCTION	16	-0.596	0.182
GO_DNA_REPLICATION_INITIATION	24	-0.547	0.183
GO_CELL_SURFACE_RECEPTOR_SIGNALING_PATHWAY_INVOLVED_IN_CELL_CELL_SIGNALING	15	-0.610	0.184
GO_PTERIDINE_CONTAINING_COMPOUND_METABOLIC_PROCESS	25	-0.537	0.186
GO_REGULATION_OF_CELL_SUBSTRATE_ADHESION	89	-0.424	0.184
GO_ORGANIC_ACID_TRANSMEMBRANE_TRANSPORT	31	-0.511	0.185
GO_CARBOHYDRATE_BIOSYNTHETIC_PROCESS	71	-0.437	0.182
GO_VESICLE_COATING	53	-0.461	0.180
GO_REGULATION_OF_RNA_STABILITY	110	-0.408	0.179
GO_CELLULAR_AMINO_ACID_BIOSYNTHETIC_PROCESS	56	-0.447	0.189
GO_REGULATION_OF_CELLULAR_PH	34	-0.493	0.187
GO_PORPHYRIN_CONTAINING_COMPOUND_METABOLIC_PROCESS	23	-0.547	0.186
GO_NUCLEAR_TRANSPORT	258	-0.374	0.185
GO_PROTEIN_LOCALIZATION_TO_MITOCHONDRION	53	-0.458	0.184
GO_RIBOSOME_ASSEMBLY	48	-0.462	0.183
GO_PYRIMIDINE_NUCLEOTIDE_METABOLIC_PROCESS	36	-0.489	0.182
GO_VITAMIN_METABOLIC_PROCESS	58	-0.448	0.180
GO_CELLULAR_GLUCOSE_HOMEOSTASIS	37	-0.480	0.180
GO_PROTEIN_TARGETING_TO_MITOCHONDRION	41	-0.472	0.179
GO_RNA_LOCALIZATION	151	-0.391	0.177
GO_REGULATION_OF_VIRAL_TRANSCRIPTION	45	-0.461	0.176
GO_ANION_TRANSMEMBRANE_TRANSPORT	70	-0.433	0.186
GO_VITAMIN_TRANSPORT	15	-0.599	0.186
GO_REGULATION_OF_TELOMERE_MAINTENANCE	53	-0.450	0.185
GO_TRANSCRIPTION_FROM_RNA_POLYMERASE_III_PROMOTER	33	-0.492	0.193
GO_TETRAPYRROLE_BIOSYNTHETIC_PROCESS	21	-0.542	0.192
GO_RIBOSOMAL_LARGE_SUBUNIT_BIOGENESIS	43	-0.471	0.195
GO_INNATE_IMMUNE_RESPONSE_ACTIVATING_CELL_SURFACE_RECEPTOR_SIGNALING_PATHWAY	72	-0.429	0.193
GO_REGULATION_OF_TELOMERE_MAINTENANCE_VIA_TELOMERE_LENGTHENING	41	-0.472	0.193
GO_NEGATIVE_REGULATION_OF_CELLULAR_RESPONSE_TO_GROWTH_FACTOR_STIMULUS	59	-0.444	0.192
GO_DICARBOXYLIC_ACID_METABOLIC_PROCESS	56	-0.444	0.197
GO_DNA_DEPENDENT_DNA_REPLICATION	79	-0.417	0.199
GO_RRNA_MODIFICATION	19	-0.544	0.201
GO_REGULATION_OF_ADHERENS_JUNCTION_ORGANIZATION	33	-0.490	0.200
GO_NCRNA_TRANSCRIPTION	72	-0.418	0.209
GO_RNA_METHYLATION	39	-0.472	0.210
GO_ORGANOPHOSPHATE_BIOSYNTHETIC_PROCESS	278	-0.361	0.213
GO_REGULATION_OF_CELL_MATRIX_ADHESION	52	-0.443	0.213
GO_NEGATIVE_REGULATION_OF_CELL_MATRIX_ADHESION	16	-0.563	0.212
GO_REGULATION_OF_PH	38	-0.475	0.212
GO_MITOCHONDRIAL_TRANSPORT	131	-0.384	0.212
GO_CARBOHYDRATE_DERIVATIVE_BIOSYNTHETIC_PROCESS	338	-0.354	0.212
GO_MATURATION_OF_5_8S_RRNA	24	-0.524	0.212
GO_CELLULAR_RESPONSE_TO_INTERFERON_GAMMA	31	-0.482	0.212
GO_MULTI_ORGANISM_LOCALIZATION	52	-0.441	0.212

GO_SNRNA_METABOLIC_PROCESS	69	-0.423	0.212
GO__DE_NOVO_PROTEIN_FOLDING	16	-0.563	0.220
GO_NIK_NF_KAPPAB_SIGNALING	62	-0.427	0.220
GO_RESPONSE_TO_ARSENIC_CONTAINING_SUBSTANCE	18	-0.554	0.226
GO_REGULATION_OF_TELOMERASE_ACTIVITY	31	-0.486	0.226
GO_PYRIMIDINE_NUCLEOTIDE_BIOSYNTHETIC_PROCESS	23	-0.521	0.225
GO_CELLULAR_AMINO_ACID_METABOLIC_PROCESS	193	-0.367	0.223
GO_REGULATION_OF_MRNA_SPLICING_VIA_SPLICEOSOME	46	-0.441	0.225
GO_CELLULAR_RESPONSE_TO_KETONE	36	-0.468	0.231
GO_CARBOHYDRATE_HOMEOSTASIS	75	-0.414	0.235
GO_REGULATION_OF_MITOCHONDRION_ORGANIZATION	140	-0.374	0.246
