# Supplementary material for: The WWOX Gene Influences Cellular Pathways in the Neuronal Differentiation of Human Neural Progenitor Cells
Source: Front Cell Neurosci. 2019 Aug 30;13:391. doi: 10.3389/fncel.2019.00391 (PMC6730490; doi:10.3389/fncel.2019.00391)
Supplement: Supplementary file 2 [file Data_Sheet_2.PDF]

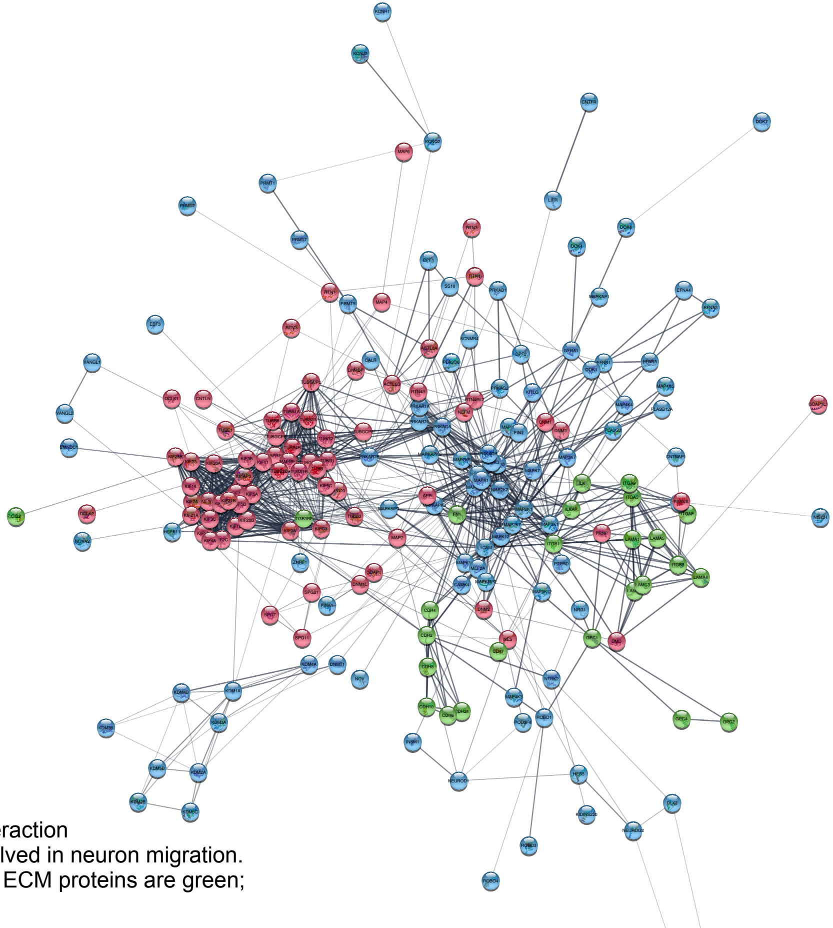

Supplementary Figure S3. Protein-protein interaction network of differentially-expressed genes involved in neuron migration. Cytoskeleton and ER are red; membrane and ECM proteins are green; signaling and chromatin remodeling are blue.
