# Supplementary material for: The WWOX Gene Influences Cellular Pathways in the Neuronal Differentiation of Human Neural Progenitor Cells
Source: Front Cell Neurosci. 2019 Aug 30;13:391. doi: 10.3389/fncel.2019.00391 (PMC6730490; doi:10.3389/fncel.2019.00391)
Supplement: Supplementary file 4 [file Data_Sheet_4.PDF]

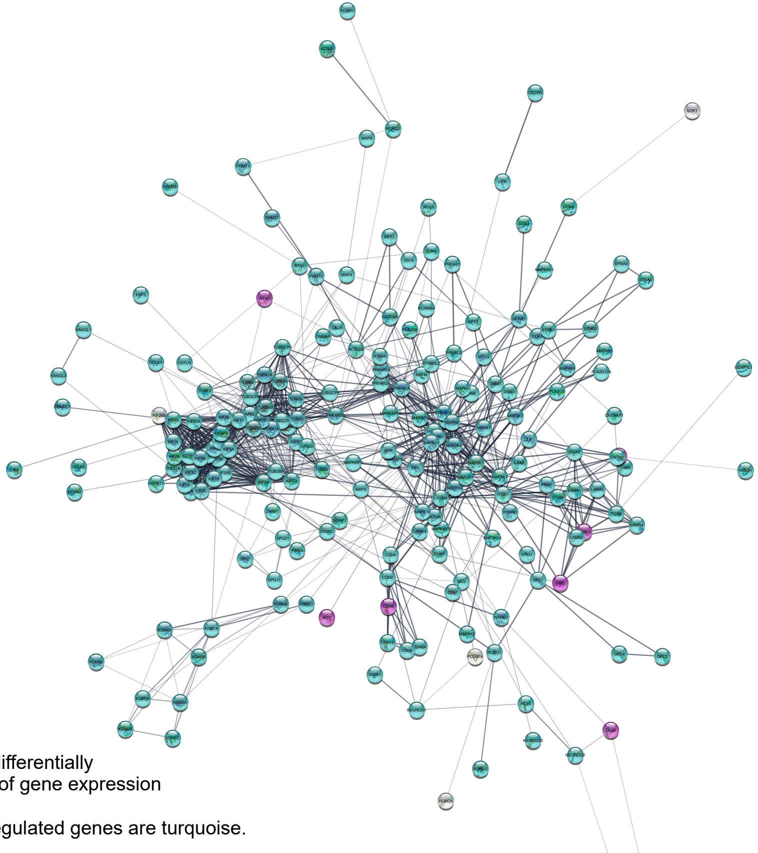

Supplementary Figure S5. Protein-protein interaction network of differentially
expressed genes involved in neuron migration. Log2 fold change of gene expression
in neurons/shWWOX vs neurons/shScrambled.
Significantly upregulated genes are magenta; significantly downregulated genes are turquoise.
